# Risk-Based Screening Tools to Optimise HIV Testing Services: a Systematic Review

**DOI:** 10.1007/s11904-022-00601-5

**Published:** 2022-02-11

**Authors:** J. J. Ong, K. Coulthard, C. Quinn, M. J. Tang, T. Huynh, M. S. Jamil, R. Baggaley, C. Johnson

**Affiliations:** 1 Central Clinical School, Monash University, Melbourne, Australia; 2 Melbourne Sexual Health Centre, The Alfred Hospital, Melbourne, Australia; 3 Department of Clinical Research, London School of Hygiene and Tropical Medicine, London, UK; 4 Global HIV, Hepatitis and STIs Programmes, World Health Organization, Geneva, Switzerland

**Keywords:** HIV, Testing, Screening tool

## Abstract

**Purpose of review:**

Effective ways to diagnose the remaining people living with HIV who do not know their status are a global priority. We reviewed the use of risk-based tools, a set of criteria to identify individuals who would not otherwise be tested (screen in) or excluded people from testing (screen out).

**Recent findings:**

Recent studies suggest that there may be value in risk-based tools to improve testing efficiency (i.e. identifying those who need to be tested). However, there has not been any systematic reviews to synthesize these studies.

**Summary:**

We identified 18,238 citations, and 71 were included. The risk-based tools identified were most commonly from high-income (51%) and low HIV (<5%) prevalence countries (73%). The majority were for “screening in” (70%), with the highest performance tools related to identifying MSM with acute HIV. Screening in tools may be helpful in settings where it is not feasible or recommended to offer testing routinely. Caution is needed for screening out tools, where there is a trade-off between reducing costs of testing with missing cases of people living with HIV.

**Supplementary Information:**

The online version contains supplementary material available at 10.1007/s11904-022-00601-5.

## Introduction

Globally, an estimated 6.0 million people living with HIV (PLHIV) remain unaware of their status, approximately 16% of the total population of PLHIV [1]. This gap in knowledge of HIV status is a significant public health problem, whereby those living with HIV who are not linked to appropriate treatment and care have higher HIV-related mortality and morbidity [2]. Finding effective and efficient ways to close this testing gap is an urgent global priority.

As nations strive to meet United Nation’s (UN) 95-95-95 testing and treatment targets—with the first target referring to having 95% of PLHIV diagnosed and aware of their status by 2025 [3]—efforts to reach the remaining undiagnosed individuals is challenging and costly. As countries successfully control the HIV epidemic, HIV positivity (or yield) may decline in parallel with increases in testing and treatment coverage, thereby increasing the cost per person diagnosed. Countries also need to make testing more efficient in light of HIV funding in low-and-middle-income countries stalling and decreasing since 2017, with further disruption of services as a result of the COVID-19 pandemic [4]. Strategic use of HIV testing services (HTS) approaches, including partner services [5], community-based testing [6••], and HIV self-testing [7, 8], focused on geographic areas, and populations with the greatest HIV burden and unmet testing need have proven effective and efficient in reaching people with undiagnosed HIV infection.

Another strategy to consider is using risk-based screening tools in HIV testing services. Risk-based screening tools typically use a set of criteria to either identify high-risk individuals for HIV testing who would not otherwise be offered a test (screen in) or exclude low-risk people from a routine offer of the test (screen out). Tools may be electronic or paper-based and can be self or provider-administered in inpatient [9, 10] and outpatient clinics [11], primary care or community settings [12]. A tool may use a combination of demographics, risk behaviours, clinical examination findings, HIV indicator conditions or presenting symptoms to ascertain the risk of HIV in the individual and suggest whether an HIV test should be offered. Currently, it is uncertain how widely used the tools are, whether tools are validated, which tools are used for what populations and how feasible and acceptable tools are to patients and providers. To date, results have varied; some programmatic implementation of screening tools suggests increased yield and positivity [6••, 13••, 14], while other reports raise concerns that these screening tools may mean people with undiagnosed HIV are not tested and missed due to limited criteria [15–17].

This study aims to use a systematic review and global survey of HTS implementers to describe which risk-based tools are used in what settings and populations, and how they perform in relation to their potential risks and benefits.

## Methods

### Search Strategy and Selection Criteria for the Systematic Literature Review

We searched Ovid MEDLINE, Ovid EMBASE, Web of Science, and Global Health Search between 1^st^ and 9th July 2020. The search terms used two key concepts: “HIV”, and “Risk assessments or screening tools.” The complete search strategy is presented in Appendix 1. The inclusion criteria were any study published from 1st January 2010 and contained primary data about using screening tools to optimise HTS. We excluded systematic literature reviews, letters, editorials, and duplicated results from the same study. The primary outcome of interest was the performance of the risk-based screening tool in terms of its sensitivity and specificity in diagnosing HIV, and the area under the receiver operating characteristic (ROC) curve [18]. Secondary outcomes included [1] external validation (i.e. testing the performance of the tool in individuals who are not the same as the development cohort); [2] characteristics of screening tools—number and type of questions, time to complete, self/provider administered, electronic/paper, self-report/clinic record based; [3] use of tool to select (screen in) or exclude (screen out) individuals from the offer of testing; [4] settings where the tool is used; [5] whether tools are being monitored; [6••] feasibility of implementing the tool, e.g. time needed to administer, impact on patient and throughput; and [7] economic evaluation.

Titles and abstracts were independently assessed for eligibility by at least two reviewers (KC, TN, MT). Another reviewer (JO) resolved any discrepancies. This systematic review has been registered at the International Prospective Register of Systematic Reviews (PROSPERO: CRD42020187838).

### Data Analysis

An extraction file was created in Microsoft Excel to collect the relevant information as per the primary and secondary outcomes outlined above. Data extraction was conducted by at least two reviewers (KC, TN, MT), and another reviewer (JO) resolved any discrepancies. The quality of each study was assessed using the appropriate critical appraisal tool from Johanna Briggs Institute [19].

### Statistical Analysis

Where available, we ranked the area under the ROC curve (AUC) from screening tools according to subpopulations (women, MSM, paediatrics) and settings (primary care, emergency department). A country with a high HIV prevalence was a national prevalence above 5%, as reported by UNAIDS [20]. All statistical analyses were performed using STATA version 16 (StataCorp. 2019. *Stata Statistical Software: Release 16*. College Station, TX: StataCorp LLC). This review is reported per Preferred Reporting Items for Systematic Reviews and Meta-Analyses (PRISMA) guidelines.

### Role of the Funding Source

The funders did not have any role in the study design; collection, analysis or interpretation of the data; writing the report or decision to submit the paper for publication.

### Patient and Public Involvement

The study did not involve any patient participation. Our preliminary findings were presented at two WHO meetings on “Optimizing HIV Testing Services Using HIV Risk Assessment Tools” (11–13^th^ November 2020 and 1^st^ June 2021) where the public could register to attend and provide feedback.

## Results

### Systematic Review Results

**Fig. 1:**
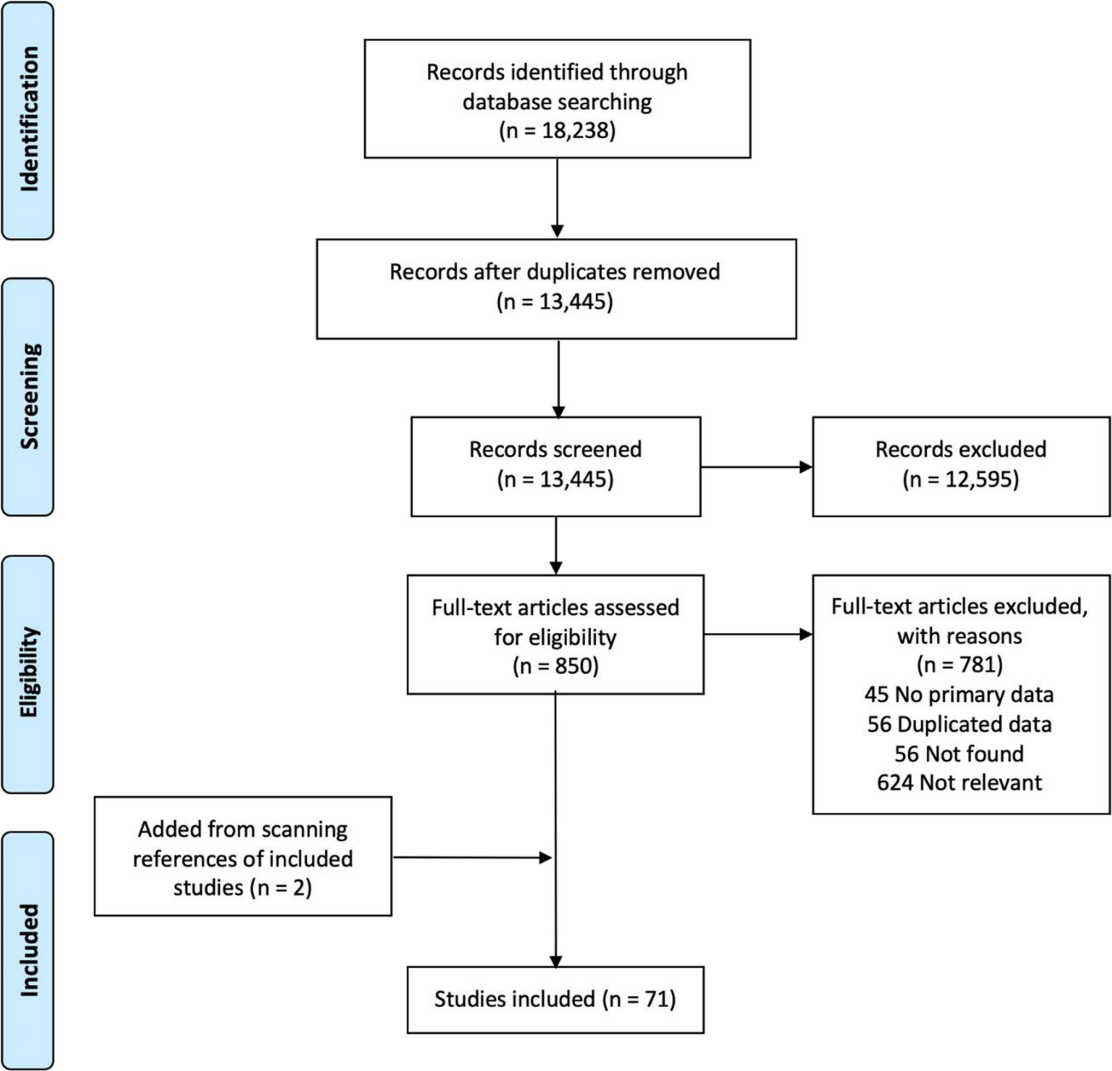
PRISMA flow diagram

The initial search identified 18,238 potential manuscripts. After removing duplicates, the titles and abstracts of 13,445 records were searched for relevance of the study objectives. We removed 12,595 records as they did not meet the study inclusion criteria. Full texts of 850 articles were assessed, with 71 included in the final analysis (Fig. 1).

**Fig. 2:**
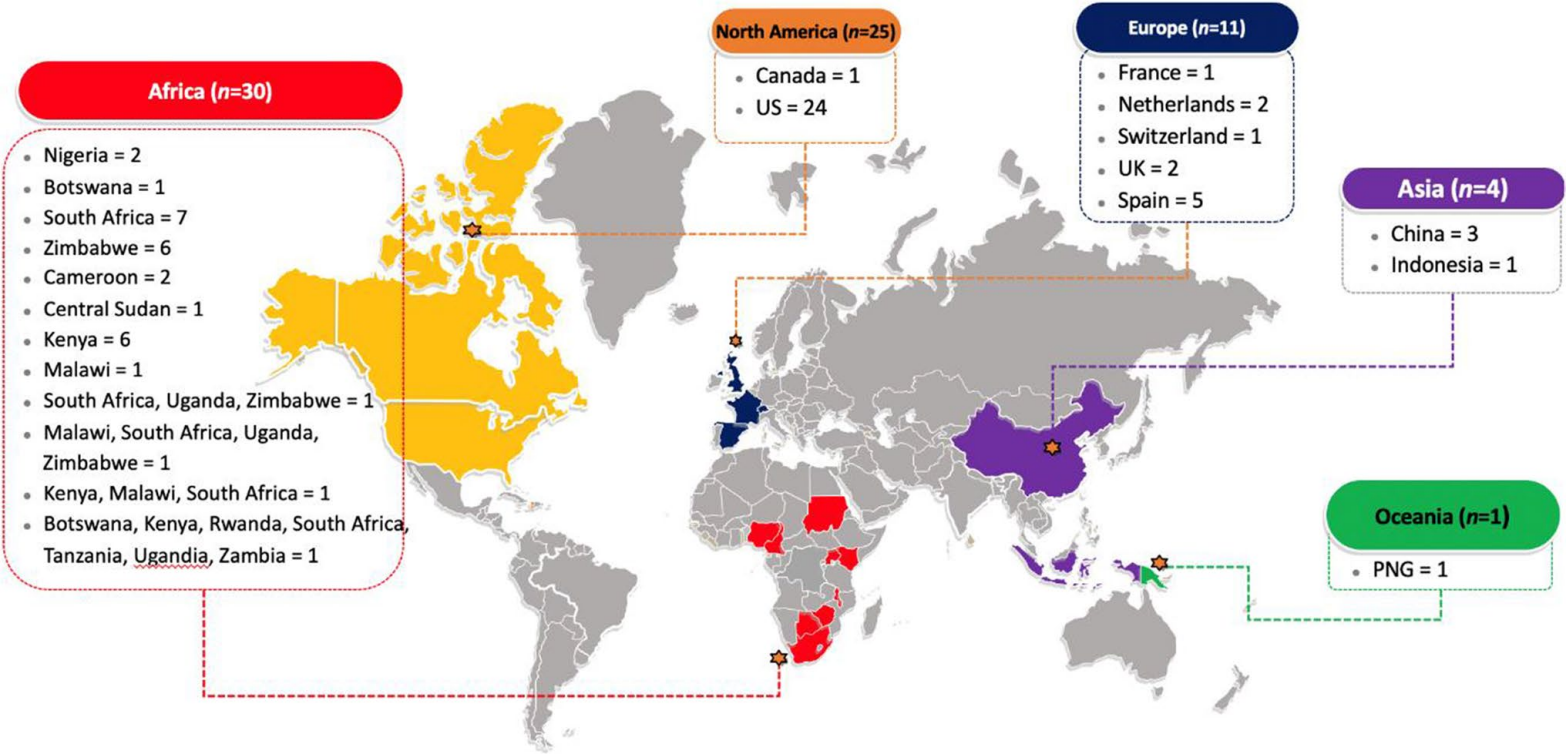
Countries of studies with an evaluation of HIV risk-based tools (*N*=71)

Figure 2 summarises the countries where the risk-based HIV screening tools were reported. Most studies arose from Africa (42%), followed by North America (35%), Europe (15%), Asia (6%), and Oceania (1%).

Table 1 summarises the characteristics of the included studies according to the country’s HIV burden. Most tools were used in high- and middle-income countries, in primary care settings, for MSM and paediatric populations, and primarily administered by the provider. The majority of risk-based screening tools were for screening in.

**Table 1:** Study characteristics, according to HIV prevalence [21]

	**Total** **(*N*=71)**	**Low HIV prevalence**^**1**^ **(*N*=52)**	**High HIV prevalence**^**1**^ **(*N*=19)**
Country income level*	***n*** **(%)**	***n*** **(%)**	***n*** **(%)**
High	36 (51)	36 (69)	0 (0)
Middle	33 (46)	15 (29)	18 (95)
Low	6 (8)	1 (2)	5 (26)
**Settings**			
Primary care	17 (24)	7 (13)	10 (53)
Hospital	13 (18)	10 (19)	3 (16)
Emergency department	11 (15)	11 (21)	0 (0)
Community	8 (11)	8 (15)	0 (0)
STI clinic	5 (7)	4 (8)	1 (5)
Antenatal or maternity ward	4 (6)	1 (2)	3 (16)
Prisons	2 (3)	2 (4)	0 (0)
**Populations**			
MSM	15 (21)	15 (29)	0 (0)
Paediatrics	14 (20)	6 (12)	8 (42)
Primary care attendees	11 (15)	11 (21)	0 (0)
Emergency department attendees	11 (15)	11 (21)	0 (0)
Women	8 (11)	1 (2)	7 (37)
Hospital inpatients	6 (8)	5 (10)	1 (5)
Adults in the community	3 (4)	1 (2)	2 (11)
STI clinic attendees	3 (4)	2 (4)	1 (5)
Incarcerated persons	2 (3)	2 (4)	0 (0)
Serodiscordant couples	2 (3)	1 (2)	1 (5)
People who inject drugs	1 (1)	1 (2)	0 (0)
Female sex workers	1 (1)	0 (0)	1 (5)
**Tool administered by**			
Patient	13 (18)	6 (12)	7 (37)
Provider	42 (52)	26 (50)	16 (84)
**Type of tool**			
Screening in	50 (70)	38 (73)	12 (63)
Screening out	21 (30)	14 (27)	7 (37)

^*^Some studies contain more than one country, so the denominator may not add up to 71. The number of missing studies is not shown

^1^High HIV prevalence is defined as ≥5%, and low HIV prevalence is defined as <5%

Table 2 summarises the risk-based tools for MSM. All tools were used to “screen in”. Most had been externally validated (13/15) and used in various settings. The tools with the highest AUC related to identifying men with acute HIV in Kenya (0.89) [22], the Netherlands (0.88) [13••], and the USA (0.85) [23]. The use of these tools helped allocate more costly diagnostics to those who are more likely to have acute HIV. Acute HIV was defined by a positive HIV-1 RNA or p24 antigen test and two negative rapid antibody tests or ELISA assays, followed by documented antibody seroconversion. Tools with the highest sensitivity were related to identifying HIV among MSM in Kenya (98% [24], 90% [22]), and MSM in the USA (84%) [25]. The tools with the highest specificity related to identifying men with acute HIV in the US (96% [23], 81% [13••]), and the Netherlands (78% [26••]). The most common domain used in the tools was the risk behaviours (Supplementary Table 1). The average number of items within a tool was 6.6 (range 4–12).

**Table 2:** Risk-based screening tools for men who have sex with men ordered by performance ordered by the area under receiver operating characteristic curve (AUC)

**Lead Author (year of publication)**	**Year(s) of data**	**Sample size**	**Country**	**Setting**	**Externally validated?**	**AUC (95% CI)**	**Sensitivity**	**Specificity**
Sanders (2015) [22]	2005-12	Unclear	Kenya	Health facilities	Yes	0.89	90%	74.1%
Lin (2018) [13••]	2007-17	757	USA	Community-based screening program	Yes	0.88 (0.84-0.91)	78.2%	81%
Lin (2018) [23]	2007-17	998	USA	Community-based screening program	Yes	0.85 (0.78-0.92)	72%	96%
Dijkstra (2017) [27]	1984-2009	1562	Netherlands	STI clinic	Yes	0.82 (0.79-0.86)	76.3% (68.2-83.2)	76.3% (75.6-77.0)
Scott (2020) [28]	2009-10	1164	US	Community	Yes	0.8	81.1%	59.6%
Wahome (2013) [29]	2005-2012	6531	Kenya	Unclear	Yes	0.79	75.3%	76.4%
Wahome (2018) [24]	2005-16	753	Kenya	Community - personal networks, sex venues	No	0.76 (0.71-0.8)	97.9%	16.9%
Smith (2012) [25]	1998-2001	7754	USA	Unclear	Yes	0.74	84%	42%
Yin (2018) [30]	2013-14	3588	China	Study clinics and community	Yes	0.71	Not reported	Not reported
Dijkstra (2020) [26••]	2003-18	1071	Netherlands	STI clinic	Yes	0.70 (0.64-0.76)	54.0%	77.9%
Hoenigl (2015) [31]	2008-2014	8326	USA	Community-based screening program	Yes	0.70 (0.63-0.78)	58%	76%
Luo (2019) [32]	2009-16	1442	China	Unclear	Yes	0.63 (0.61-0.66)	Nor reported	Not reported
Jones (2018) [33]	2010-14	562	USA	Recruited from venue-based time-space sampling and via Facebook ads	Yes	HIRI: 0.62 (0.52-0.72) Menza: 0.51 (0.41-0.60) SDET: 0.55 (0.44-0.66)	HIRI: 62.5% (43.7-78.9) Menza: 62.5% (43.7-78.9) SDET: 25% (11.5-43.4)	HIRI: 56.7% (52.4-61.0) Menza: 41.1% (36.9-45.5) SDET: 83.9% (80.5-87.0)
Yun (2019) [34]	2009-16	999	China	VCT in hospital, recruitment from community	Yes	0.6 (0.45-0.74)	Not reported	Not reported
Beymer (2017) [35]	2009-14	9481	USA	LGBT Centre	No		75%	50%

*LGBT* lesbian, gay, bisexual and transgender; *STI* sexually transmitted infection; *VCT* voluntary counselling and testing; *95% CI* 95% confidence interval

Table 3 summarises the risk-based tools for the paediatric population. Most studies were for 'screening out' (67%, 8/12). A minority had been externally validated (6/13). Only two studies reported AUCs (0.73 [36], 0.65 [6••]); both were generally lower than most tools used for MSM. The tools with the highest sensitivity were for prioritising children for HIV testing at birth in Botswana (100% [37]), hospitalised paediatric patients in Papua New Guinea (96% [38]) and Malawi (84% [39]). The tools with the highest specificity were for targeting hospitalised paediatric patients in Central Sudan (96% [40]) and Zimbabwe (88% [41]). The most common domain used in the tools was symptoms and signs (Supplementary Table 2). The average number of items within a tool was 5.2 (range 4–9).

**Table 3:** Risk-based screening tools for paediatric population ordered by the area under receiver operating characteristic curve (AUC)

**Lead Author (year of publication)**	**Year of data**	**Sample size**	**Country**	**Setting**	**External validation?**	**AUC**	**Sensitivity**	**Specificity**
Bandason (2016) [36]*	2013-14	9568	Zimbabwe	Primary care	Yes	0.73 (0.72-0.75)	80.4% (76.5-84.0)	66.3% (65.3-67.2)
Bandason (2018) [6••]*	2015	5384	Zimbabwe	Community	Unclear	0.65 (0.60-0.72)	56.3% (44-68.1)	75.1% (73.9-76.3)
Ibrahim (2018) [37]*	2015-16	2303	Botswana	Hospital maternity wards	No	Not reported	100%	Not reported
Allison (2011) [38]	2007-08	487	PNG	Hospital	Yes	Not reported	96.3%	25%
Moucheraud (2018) [39]	2016-17	8602	Malawi	Inpatient paediatric ward	Yes	Not reported	84.4%	39.6%
Bandason (2015) [42]*	2015	6102	Zimbabwe	Primary health centre	Yes	Not reported	80% (75-85)	66% (95% CI 64-67)
Du Plessis (2019) [43]*	2014-16	1759	South Africa	Hospital maternity wards	No	Not reported	80%	64%
Ferrand (2011) [44]*	2011	506	Zimbabwe	Primary care	Yes	Not reported	74% (64-82)	80% (71-87)
Mafaune (2020) [45]*	2018-19	1970	Zimbabwe	Health facility (Antenatal)	No	Not reported	62.1%	87.2%
Bandason (2018) [6••]*	2015	5384	Zimbabwe	Unclear	Unclear	Not reported	56.3% (44-68.1)	75.1% (73.9-76.3)
Nathoo (2012) [41]	2012	355	Zimbabwe	Medical paediatric wards	No	Not reported	43%	88%
Abbas (2010) [40]	2007-08	127	Central Sudan	Hospital	Unclear	Not reported	WHO-CCD 16.7% (0.4-64.1), B-CCD 33.3% (4.3-77.7), MB-CCD 66.7% (22.3-95.7)	96% (90.1-98.9), 88% (80-93.6) 74% (64.3-82.3)

^*^Screening out tools

Table 4 summarises the risk-based tools for targeting women. All tools were used to “screen in”. A majority had been externally validated (5/8). The tools with the highest AUC were used for targeting pregnant women in Kenya (0.84) [46], and sexually active women in South Africa (0.75 [47] and 0.73 [14]). The tools with the highest sensitivity were for targeting women in South Africa (96% [48]), women in South Africa/Uganda/Zimbabwe (91% [49]) and Malawi/South Africa/Uganda/Zimbabwe (90%) [50]. The tools with the highest specificity were for targeting sexually active women (18–30 years old) in South Africa (84% ) [48], sexually active women (18–24 years old) in South Africa (84%) [51] and sexually active women (18–35 year old) in South Africa (71%) [51]. The most common domain used in the tool was risk behaviours (Supplementary Table 3). The average number of items within a tool was 6 (range 4–7).

**Table 4:** Risk-based screening tools for women ordered by the area under receiver operating characteristic curve (AUC)

**Author**	**Year**	**Sample size**	**Country**	**Setting (target group)**	**External validation?**	**AUC**	**Sensitivity**	**Specificity**
Pintye (2017) [46]	2011-14	1304	Kenya	Antenatal clinics (pregnant women)	Yes	0.84 (95%CI 0.72-0.95) 0.76 (0.67-0.85) – simplified score	Not reported	Not reported
Wand (2018) [47]	2002-12	8982	South Africa	Part of trial (sexually active 16+)	Yes	0.75 - development 0.71 - validation	83% (development) 80% (validation)	33% (development) 32% (validation)
Wand (2012) [14]	2003-06	1485	South Africa	Unclear (women 18-49)	Yes	0.73 (0.66-0.79) - development 0.79 (0.70-0.81) - validation	88% (development) 90% (validation)	32% (development) 36% (validation)
Balkus (2016) [49]	2009-11	5029	South Africa, Uganda, Zimbabwe	Part of trial (women 18-40)	Yes	0.67 (0.64-0.70) – development 0.7 (0.65-0.75) – validation with HPTN035 0.58 (0.51-0.65) – validation with FEM-PrEP	91% (development) 84% (HPTN035) 83% (FEM-PrEP)	38% (development) 46% (HPTN035) 31% (FEM-PrEP)
Balkus (2016) [50]	2016	1269	Malawi, South Africa, Uganda, Zimbabwe	Part of trial (women 18-40) to externally validate the tool.	Yes	0.66 (0.6-0.73)	90%	35%
Burgess (2018) [52]	2018	444	South Africa	Unclear (women 18-40)	Unclear	0.66 (0.54-0.74) – overall 0.69 (0.6-0.78) - age <25 0.49 (0.3-0.63) – age >25	64% (overall) 78% (age <25) 58% (age >25)	57% (overall) 49% (age <25) 38% (age >25)
Peebles (2018) [51]	2015-18	5573	South Africa	A diverse range of settings across five provinces (women 18-35)	Yes	0.64 (0.6-0.67) – age 18-24 0.68 (0.62-0.73) – age 25-35 0.61 (0.58-0.65) – using VOICE score [49]	48.6% (age 18-24) 78.6% (age 25-35)	70.8% (age 18-24) 42.7% (age 25-35)
Burgess (2017) [48]	2011-14	1115	South Africa	9 South African sites (sexually active, 18-30)	No	0.56 (0.5-0.62)	96% (Risk score >3) 84% (Risk score >5)	84% (Risk score >5) 23% (Risk score >5)

The risk-based tools are provided for emergency department attendees (Supplementary Tables 4 and 5), primary care attendees (Supplementary Tables 6 and 7), hospital inpatients (Supplementary Tables 8 and 9), adults in the community (Supplementary Tables 10 and 11), STI clinic attendees (Supplementary Tables 12 and 13), incarcerated persons (Supplementary Tables 14 and 15), serodiscordant couples (Supplementary Tables 16 and 17), and people who inject drugs (Supplementary Tables 18 and 19). The risk of bias assessments are provided in Supplementary Tables 20-24.

## Discussion

This systematic review highlights the use of HIV screening tools in both high and low HIV prevalence settings. We found that published tools were mostly used to screen in and prompt testing for those who may be missed otherwise, primarily from high or middle-income countries and administered predominantly in primary care settings. Screening out tools were mainly used in neonatal or paediatric settings. We found that the tools with the highest accuracy existed for identifying acute HIV infections among MSM. Caution should be exercised when using risk-based screening tools for other populations, as we found variable performance depending on their setting. In low HIV prevalence settings where HIV testing is not routinely offered, there is benefit in using the tools to screen in those with a greater risk of HIV acquisition. However, there is a trade-off in using these tools in high HIV prevalence settings and/or among key populations. Using these tools to reduce testing offer in these contexts risk missing undiagnosed people living with HIV.

There was some evidence on the potential risks and benefits of tools that would screen out and not test individuals. The systematic literature found several studies to reduce testing volume with mixed results. There seems to be value in using tools to target expensive acute HIV screening tests among MSM [53]. In low HIV prevalence settings, these screening out tools have high negative predictive values (NPV): a tool for emergency department and primary care attendees in Spain (100% NPV) [54], a tool to identify children living with HIV in a community setting in Zimbabwe (99% NPV) [6••], and a tool to identify adolescents attending primary care in Zimbabwe (100% NPV) [53]. Targeted screening of infants compared with universal testing in Botswana could lower costs of testing [37]. However, studies show that screening out tools for newborn testing could miss 1 in 5 newborns in South Africa [43] and 2 in 5 in Zimbabwe [45]. Further, the use of targeted screening tools in US emergency departments [55] or among US veterans [16] would still miss cases of people living with HIV. In some instances, screening out tools did not significantly reduce testing volume nor increase positivity rates [56, 57]. Together, the evidence suggests that providers must be cautious in using screening out tools depending on the target population, as missing people with HIV who would otherwise be tested could undermine efforts to achieve ambitious 95-95-95 targets.

There could be several benefits to using risk-based screening in tools. First, screening tools evaluated were generally effective in identifying people who had a higher likelihood of HIV, thereby improving allocative efficiency by targeting limited resources to those with higher risks of HIV acquisition [58], prioritising patients who need expensive tests (e.g. for identifying acute HIV) [22, 23, 26••] or prioritising individuals in need of more expensive prevention methods like PrEP [25, 28, 59–62, 63••, 64]. However, several studies underscore the importance of using locally validated tools as the performance of tools could differ according to race [65] or age [51]. Second, we found that in settings where patients may not be forthcoming about risk factors or where clinicians are not likely to ask, the implementation of risk-based tools prompted the offer of testing and improved HIV testing uptake [44, 66, 67]. Except for three papers [17, 30, 54], no other study discussed how privacy and confidentiality were maintained when administering the screening tool. Third, most screening tools were simple enough to allow their use by non-professional health workers, such as lay counsellors or self-assessments [39]. There have been many innovations with virtual interventions, particularly during COVID-19, such as online HIV self-testing which included self-risk assessment tools [68]. Using screening in tools could further improve the efficiencies of decentralised HIV testing services by enabling the training of lay providers, peers, and clients to use these tools in a range of contexts and settings to focus HIV testing outreach. Last, risk-based screening tools may be more cost-effective than routine testing in some settings. An economic evaluation from the USA reported that targeted testing compared with routine testing in clinics, hospitals, and community-based organisations was more cost-effective per diagnosis and per transmission averted [69]. In addition, targeted testing compared with testing patients suspected to have symptomatic HIV in US emergency departments was found to be cost-saving [70]. The cost per new diagnosis in a primary care setting in Spain was €129 compared with €2001 for routine testing [71].

There may be potential harms to using HIV risk-based screening in tools. First, the screening questions that seek to identify people with high HIV risks could potentially be stigmatising and reduce testing uptake. For example, a study in Indonesia used self-reported injecting drug use as part of the risk-based screening tool among incarcerated persons [15]. This led to under-reporting of injecting drug use and 1 in 3 eligible people declining to test [15]. Second, given suboptimal sensitivity for some tools, there is potential for missing cases of people living with HIV [26••]. This is particularly important for tools used for antenatal or paediatric populations where the consequences of missing an HIV case outweighs any benefits of risk-based HIV testing [6••, 41, 43]. Therefore, universal opt-out testing should be standard practice in settings with serious consequences for missing a case. Third, there are resource implications when implementing risk-based screening as there is a need for regular external validation of tools to local contexts to ensure its expected accuracy, correct items to include, optimal length, acceptability by providers and patients, and the impact on patient flow [26••, 54, 72]. This ongoing evaluation and monitoring could impact the feasibility of implementing these tools.

Overall, an ideal HIV risk-based screening tool should be accurate, preferably with an area under the curve (AUC) of over 0.8 [18]. Tools should be externally validated to account for variations in HIV epidemiological profiles (even within the same country). Further, as risk factors may change over time, regular evaluations every few years may be necessary to ensure the tools continue to perform optimally and are appropriately adapted to local contexts. Tools must be reliable and ideally be based on objective measures rather than self-reported behaviours, which might be inaccurate if risk items are stigmatising or challenging to measure. This includes careful evaluation of the language construction of risk-based tools such that they are culturally appropriate for each setting. Tools should be administered in a private setting to maintain confidentiality. Finally, tools must be feasible to implement using simple questionnaires acceptable to the provider and patient and do not adversely affect the clinic flow.

The strength of this research is that we comprehensively searched the literature to provide an overview of HIV-risk-based tools. We identified tools with high accuracy targeting different populations, and explored the advantages and disadvantages of implementing such tools. There are limitations to the study. We did not include any non-English language data, which may lead to selection bias. There would be a possibility of publication bias if screening tools that performed poorly were not published.

## Conclusion

As evidence continues to accumulate for HIV risk-based tools, we strongly encourage considerations on the role of screening in tools in settings where the routine offer of testing is not feasible or recommended, and how these could be adapted to self-assessment, targeted outreach, distribution of self-tests, and incorporated into virtual interventions for HIV testing. Caution must be exercised for screening out tools, where there is a trade-off between reducing costs of testing with missing cases of people living with HIV. We also encourage programmes to construct, adapt and regularly evaluate the implementation of any HIV risk-based screening tools to ensure they do not undermine progress toward the 95-95-95 targets. Further data will also be needed to evaluate the cost-effectiveness of HIV risk-based screening tools and assess any differences in linkage to care for people tested using risk-based tools.

## Supplementary Information

ESM 1 (DOCX 233 kb)

## Data Availability

All relevant data are presented in the manuscript and online supplementary materials. Any further details can be obtained by contacting the corresponding author.
