# Supplementary material for: Risk-Based Screening Tools to Optimise HIV Testing Services: a Systematic Review
Source: Curr HIV/AIDS Rep. 2022 Feb 11;19(2):154–65. doi: 10.1007/s11904-022-00601-5 (PMC8832417; doi:10.1007/s11904-022-00601-5)
Supplement: Supplementary file 1 — (DOCX 233 kb) [file 11904_2022_601_MOESM1_ESM.docx]

**APPENDIX 1 – Search Strategy**

**Medline Ovid – 9th July 2020**

1. (HIV or "human immunodeficiency virus" or "acquired immunodeficiency" or "acquired immune deficiency" or AIDS or "HIV infect*" or "acquired immunodeficiency syndrome" or "acquired immune deficiency syndrome" or "acquired immuno-deficiency syndrome" or "acquired immune-deficiency syndrome" or "HIV-infected" or "HIV-positive" or "HIV/AIDS" or "HIV-1" or "HIV-2" or hiv1 or hiv2 or "HIV diagnos*" or serodiagnos* or "HIV acquisition" or "acquir* HIV").ti,ab.

2. *HIV Infections/di or *Acquired Immunodeficiency Syndrome/di or *HIV Seropositivity/di or *AIDS Serodiagnosis/ or *HIV Infections/pc

3. 1 or 2

4. (screen* or test or tests or tested or testing).ti,ab.

5. *Mass Screening/mt or *Diagnostic Tests, Routine/mt

6. (Risk* or high-risk).ti,ab.

7. *Risk Assessment/ or *Risk Factors/ or *Risk-taking/ or *Risk/

8. 4 or 5 or 6 or 7

9. (tool* or assess* or scoring or score or validation or predict* or profil* or stratification or decision* or "artificial intelligence" or algorithm* or calculat* or detect* or comput* or question* or scale* or evaluat* or target* or strateg* or performance or sensitivity or specificity).ti.

10. *Decision Support Techniques/ or * "Sensitivity and Specificity"/

11. 9 or 10

12. 3 and 8 and 11

13. Animals/

14. 12 not 13

15. limit 14 to yr="2010 - 2020"

11367 results

**EMBASE Ovid – 9th July 2020**

1. (HIV or "human immunodeficiency virus" or "acquired immunodeficiency" or "acquired immune deficiency" or AIDS or "HIV infect*" or "acquired immunodeficiency syndrome" or "acquired immune deficiency syndrome" or "acquired immuno-deficiency syndrome" or "acquired immune-deficiency syndrome" or "HIV-infected" or "HIV-positive" or "HIV/AIDS" or "HIV-1" or "HIV-2" or hiv1 or hiv2 or "HIV diagnos*" or serodiagnos* or "HIV acquisition" or "acquir* HIV").ti.

2. (screen* or test or tests or tested or testing or risk* or high-risk).ti.

3. (tool* or assess* or scoring or score or validation or predict* or profil* or stratification or decision* or artificial intelligence or algorithm* or calculat* or detect* or comput* or question* or scale* or evaluat* or target* or strateg* or performance or sensitivity or specificity).ti.

4. 1 and 2 and 3

5. animal/

6. 4 not 5

7. limit 6 to yr="2010 - 2020"

3212 results

**Web of Science – 1st July**

Indexes=SCI-EXPANDED, SSCI, A&HCI, CPCI-S, CPCI-SSH, BKCI-S, BKCI-SSH, ESCI, CCR-EXPANDED, IC Timespan=2010-2020

1. ti=(HIV or "human immunodeficiency virus" or "acquired immunodeficiency" or "acquired immune deficiency" or AIDS or "HIV infect*" or "acquired immunodeficiency syndrome" or "acquired immunedeficiency syndrome" or "acquired immuno-deficiency syndrome" or "acquired immune-deficiency syndrome" or "HIV-infected" or "HIV-positive" or "HIV/AIDS" or "HIV-1" or "HIV-2" or hiv1 or hiv2 or "HIV diagnos*" or serodiagnos* or "HIV acquisition" or "acquir* HIV")

2. ti=(screen* or test or tests or tested or testing or risk* or high-risk)

3. ti=(tool* or assess* or scoring or score or validation or predict* or profil* or stratification or decision* or artificial intelligence or algorithm* or calculat* or detect* or comput* or question* or scale* or evaluat* or target* or strateg* or performance or sensitivity or specificity)

4. #3 AND #2 AND #1

3550 results

**Global Health search – 2nd July 2020**

title:(HIV or "human immunodeficiency virus" or "acquired immunodeficiency" or "acquired immune deficiency" or AIDS or "HIV infect*" or "acquired immunodeficiency syndrome" or "acquired immunedeficiency syndrome" or "acquired immuno-deficiency syndrome" or "acquired immune-deficiency syndrome" or "HIV-infected" or "HIV-positive" or "HIV/AIDS" or "HIV-1" or "HIV-2" or hiv1 or hiv2 or "HIV diagnos*" or serodiagnos* or "HIV acquisition" or "acquir* HIV") AND title:(screen* or test or tests or tested or testing or risk* or high-risk) AND title:(tool* or assess* or scoring or score or validation or predict* or profil* or stratification or decision* or artificial intelligence or algorithm* or calculat* or detect* or comput* or question* or scale* or evaluat* or target* or strateg* or performance or sensitivity or specificity) AND yr:[2010 TO 2020]

109 results

**TABLE OF CONTENTS FOR SUPPLEMENTARY TABLES**

**Supplementary Table 1 Domains used in HIV-risk based tools for men who have sex with men**

**Supplementary Table 2 Domains used in HIV-risk based tools for paediatric populations**

**Supplementary Table 3 Domains used in HIV-risk based tools for women**

**Supplementary Table 4 Risk-based screening tools for Emergency Department attendees**

**Supplementary Table 5 Domains covered by tools used for Emergency Department attendees**

**Supplementary Table 6 Risk-based screening tools for Primary care attendees**

**Supplementary Table 7 Domains covered by tools used for Primary care attendees**

**Supplementary Table 8 Risk-based screening tools for Hospital inpatients**

**Supplementary Table 9 Domains covered by tools used for Hospital inpatients**

**Supplementary Table 10 Risk-based screening tools for Adults in the community**

**Supplementary Table 11 Domains covered by tools used for Adults in the community**

**Supplementary Table 12 Risk-based screening tools for STI clinic attendees**

**Supplementary Table 13 Domains covered by tools used for STI clinic attendees**

**Supplementary Table 14 Risk-based screening tools for incarcerated persons**

**Supplementary Table 15 Domains covered by tools used for incarcerated persons**

**Supplementary Table 16 Risk-based screening tools for serodiscordant couples**

**Supplementary Table 17 Domains covered by tools used for serodiscordant couples**

**Supplementary Table 18 Risk-based screening tools for people who inject drugs**

**Supplementary Table 19 Domains covered by tools used for people who inject drugs**

**Supplementary Table 20 Risk of bias assessment using Johanna Brigg's critical appraisal tool for cross-sectional studies** ^1^

**Supplementary Table 21 Risk of bias assessment using Johanna Brigg's critical appraisal tool for case-control studies** ^1^

**Supplementary Table 22 Risk of bias assessment using Johanna Brigg's critical appraisal tool for cohort studies** ^1^

**Supplementary Table 23 Risk of bias assessment using Johanna Brigg's critical appraisal tool for quasi-experimental studies** ^1^

**Supplementary Table 24 Risk of bias assessment using Johanna Brigg's critical appraisal tool for randomised controlled studies** ^1^

**Supplementary Table 1 Domains used in HIV-risk based tools for men who have sex with men**

| **Lead Author (Year of Publication)** | **Number of questions** | **Demographics** | **Symptoms and signs** | **Indicator conditions** | **Risk behaviours** |
| --- | --- | --- | --- | --- | --- |
| Lin (2018)^2^ | 7 |  | fever, lymphadenopathy, weight loss |  | last 3 months: gonorrhoea, > 3 partners, >5 partners, condomless receptive anal sex |
| Lin (2018)^3^ | 3 |  | fever, myalgia, weight loss 14 days before testing encounter |  |  |
| Dijkstra (2017)^4^ | 7 |  | fever, lymphadenopathy, oral thrush, weight loss |  | Last 6 months: gonorrhoea, condomless receptive anal sex, >5 partners |
| Scott (2020)^5^ | 12 | age, Black, Latino |  |  | number of condomless receptive anal sex with HIV+ or unknown, number of receptive anal sex with condoms with HIV+ or unknown, number of condomless insertive anal sex with HIV+ or unknown, 1 HIV neg partner only, heavy alcohol use, methamphetamine use, popper use, Gonorrhoea/syphilis/chlamydia diagnosis |
| Wahome (2013)^6^ | 6 | age <30 | fever, diarrhoea, fatigue, symptomatic STI |  | discordant HIV test result |
| Sanders (2015)^7^ | 7 | age | fever, diarrhoea, fatigue, body pains, sore throat, genital ulcer |  |  |
| Wahome (2018)^8^ | 5 | age |  |  | sex of partner in past 3 months, sex exposure and protection with condoms in the past week, RAI in past 3 months, group sex in past 3 months |
| Smith (2012)^9^ | 7 | age |  |  | In last 6 months: total number of male partners, number of male partners who have HIV, number of condomless receptive anal sex with any HIV status partner, times had condomless insertive anal sex with HIV+ partner, used amphetamines, used poppers |
| Yin (2018)^10^ | 12 | Beijing resident, number of years living in Beijing |  |  | Drug addiction in past 3 months, alcohol before recent sex, years since first sex, lifetime male sex partners, number of male partners in last 3 months, number of receptive anal sex in last 3 months, number of insertive anal sex in last 3 months, recent anal/vaginal sex with a female partner, current syphilis, % of condom use during receptive anal sex |
| Dijkstra (2020)^11^ | 4 |  |  |  | In the preceding 6 months: condomless receptive anal sex with MSM +ve for HIV, condomless receptive anal sex with 3 or more partners, 5 or more male partners, bacterial STI diagnosis |
| Hoenigl (2015)^12^ | 4 |  |  |  | In the preceding 12 months: condomless receptive anal sex with HIV+ MSM, condomless receptive anal sex with >=5 male partners, >=10 male partners, bacterial STI |
| Luo (2019)^13^ | 9 |  |  |  | In last 6 months: number of homosexual partners (anal/oral sex), HIV+ homosexual partners, condomless anal sex with man, commercial male sex, STI diagnosis, sex role during anal sex with man, recreational drug use, group sex with men |
| Jones (2017)^14^ - HIRI | 6 | age |  |  | In last 6 months: total number of male partners, total number of HIV+ partners, number of episodes of condomless receptive anal sex, number of episodes of condomless insertive anal sex with HIV+ partner, amphetamine or popper use |
| Jones (2017)^14^ – MENZA | 4 |  |  |  | Gonorrhoea/chlamydia/syphilis diagnosis at baseline, methamphetamine or popper use in the past 6 months, number of male partners in the past 12 months, condomless anal sex with a serodiscordant partner |
| Jones (2017)^14^ – SDET | 4 |  |  |  | In the past 12 months: 10 or more partners, any condomless receptive anal sex and at least 5 partners, any condomless receptive anal sex with an HIV+ partner, gonorrhoea/chlamydia/syphilis diagnosis |
| Yun (2019)^15^ | 4 |  |  |  | Main venue for finding male sexual partners, condomless receptive anal sex, condomless insertive anal sex, used rush poppers |
| Beymer (2017)^16^ | 11 | race, age group, race of the last partner, age of last sex partner |  |  | History of chlamydia/gonorrhoea/syphilis, receptive anal sex at last sex, number of sex partners in last 3 months, intimate partner violence, use of ecstasy in last 12 months, use of methamphetamines in last 12 months, use of inhaled nitrates in last 12 months |

**Supplementary Table 2 Domains used in HIV-risk based tools for paediatric populations**

| **Lead Author (year of publication)** | **Number of questions** | **Demographics** | **Symptoms and signs** | **Indicator conditions** | **Risk behaviours** |
| --- | --- | --- | --- | --- | --- |
| Abbas (2010)^17^ | 9 |  | Major: weight loss or abnormally slow growth; chronic diarrhoea > 1 month; prolonged fever > 1 month Minor: generalised lymphadenopathy; oropharyngeal candidiasis; recurrent common infections; persistent cough; generalised dermatitis; confirmed maternal HIV infection |  |  |
| Allison (2011)^18^ | 4 |  | low weight for age, persistent fever (for >=1 month), enlarged lymph nodes (neck, axillae, groin) | oral candidiasis |  |
| Allison (2010)^19^ | 4 |  | Persistent fever, lymphadenopathy, underweight for age, oral candidiasis |  |  |
| Bandason (2016)^20^* | 4 | previous hospitalisation, orphanhood | poor health status, recurring skin problems |  |  |
| Bandason (2018)^21^* | 4 | previous hospitalisation, orphanhood | poor health status, recurring skin problems |  |  |
| Bandason (2015)^22^* | 4 | previous hospital admission, orphanhood | poor health status, recurring skin problems |  |  |
| Du Plessis (2019)^23^* | 4 | mother received cART <4 weeks before delivery, mother with VL>=1000; (4 risk model included infant small for gestational age) | symptomatic newborn |  |  |
| Ferrand (2011)^24^* | 5 | admitted to hospital, one or more natural parents died | recurring skin problems, poor health affected ability to function in daily life in past 3 months, symptoms and/or signs of STI? |  |  |
| Ibrahim (2018)^25^* | 6 |  |  |  | <8 w ART in preg, last known CD4 <250, last known VL >400, poor maternal ART adherence, lack of maternal ZDV in labour, lack of infant PEP |
| Kakanfo (2018)^26^* | 4 | previous hospitalisation, orphanhood, | poor health status, recurring skin problems |  |  |
| Mafaune (2020)^27^* | 5 |  |  |  | Mother diagnosed with HIV in labour and delivery, mother start ART after 32w gestation, maternal VL >1000 in 3rd trimester, mother seroconvert during pregnancy, mother not adhering to ART during pregnancy |
| Moucheraud (2018)^28^ | 6 | previous admission to the hospital, one or both natural parents died | recurring skin problems, sicker more often than other children in last 3 m, freq ear discharge, shorter or smaller than others in the same age group |  |  |
| Nathoo (2012)^29^ | 8 |  | Diarrhoea, vomiting, rash, ear discharge, parotid swelling, weight loss | Parent TB |  |

* Screening out tools

**Supplementary Table 3 Domains used in HIV-risk based tools for women**

| **Lead Author (year of publication)** | **Number of questions** | **Demographics** | **Symptoms and signs** | **Indicator conditions** | **Risk behaviours** |
| --- | --- | --- | --- | --- | --- |
| Balkus (2016)^30^ | 7 | age, married or living with husband/primary partner, partner provides financial/material support |  |  | alcohol use in last 3 months, partner has other sexual partners, curable STI at baseline (CT, NG, TV, TP), HSV-2 serostatus |
| Balkus (2016)^31^ | 4 | age |  |  | partner has other partners, alcohol use in last 3 m, curable STI |
| Burgess (2018)^32^ | 7 | age, unmarried/not living with partner, partner does not provide financial support |  |  | primary partner has other partners, alcohol use in past 3 months, has STI at baseline, HSV2 status |
| Burgess (2017)^33^ | 7 | age, living with partner, partner provides financial support | curable STI, HSV2 positive |  | alcohol use in last 3 months, partner has other partners |
| Peebles (2020)^34^ | 7 | HIV prevalence |  |  | weekly alcohol, number of sex partners in previous 3 months, partners have sex with others, condom use |
| Pintye (2017)^35^ | 5 |  |  |  | number of lifetime sexual partners, male partner HIV status, syphilis status, BV status, candidiasis status |
| Wand (2012)^36^ | 4 |  | genital epithelial disruption, genital discharge |  | Lifetime sexual partners, behaviour risk, cohabiting with a sex partner |
| Wand (2018)^37^ | 7 | <25 yo, single/not cohabiting, parity (<3), age at sexual debut (<16) |  |  | >=3 sexual partners in past 3 months, using injectables, diagnosis with STIs |

**Supplementary Table 4 Risk-based screening tools for Emergency Department attendees**

| **Lead Author (year of publication)** | **Year of data** | **Sample size** | **Country** | **Setting (target group)** | **External validation?** | **AUC** | **Sensitivity** | **Specificity** |
| --- | --- | --- | --- | --- | --- | --- | --- | --- |
| Lyons (2013)^38^* | 2008-2010 | 4692 | US | ED (no known HIV, 18-64) | No |  |  |  |
| Dowdy (2011)^39^* | 2008-2009 | 3766 | USA | ED (no known HIV infection or documented HIV test in the preceding 6 months) | No |  |  |  |
| Hsieh (2018)^40^* | 2015-2016 | 7931 | USA | ED (>=18, not already HIV infected, not critically ill, no altered mentation) | Yes |  |  |  |
| Hsieh (2014)^41^* | 2005-2009 | 15184 | USA | ED (<64) | Yes | AUC 0.75 (0.71-0.79) |  |  |
| Haukoos (2015)^42^* | 2008-2010 | 4830941 | USA | ED, hospitals, outpatient clinics, STI+HIV counselling and testing sites, CBOs, blood banks, plasma centres, correctional facilities (>= 13) | Yes | AUC 0.77 (0.77-0.77) |  |  |
| Haukoos (2013)^43^* | 2010-2011 | 28506+29510 | USA | ED (clinically stable, >= 13, able to provide consent, not a prisoner or detainee, not a victim of sexual assault, have not sought care as a result of occupational exposure to HIV, not self-identified as being infected with HIV) | Unclear |  |  |  |
| Haukoos (2017)^44^* | 2017 | 76561 | USA | ED (not critically ill/ mentally altered, not HIV+, >=16) | Yes |  |  |  |
| Gillet (2018)^45^* | 2015 | 160 | Switzerland | ED (18-75, clinically stable) | No |  |  |  |
| Haukoos (2012)^46^* | 1996-2008 | 92635 (derivation), 22983 (validation) | USA | ED, but derivation set from STD clinic (Derivation population = 13, attended STD clinic between 1996-2008) | Yes. | Derivation (AUC 0.86, 0.84-0.88); Validation (AUC 0.75, 0.70-0.78) |  |  |
| Elias (2016)^47^ | 2012-2013 | 5329 | Spain | ED and primary care centre (18-60) | No |  | 100% | 49% |
| Ayerbe (2016)^48^ | 2016 | 5329 | Spain | ED and primary care centres (18-60) | No | RE&CI-Q: Sens 100%, spec 49%, PPV 0.8%, NPV 100%. Denver HIV risk score: Sens 72.7%, spec 60.4%, PPV 0.76%, NPV 99.8%; HIV indicator diseases across Europe Study (HIDES): Sens 91%, spec 74.4%, PPV 1.4%, NPV 99.9% | RE&CI-Q = 100% Denver HIV risk score = 72.7% HIDES = 91% | RE&CI-Q = 49% Denver HIV risk score = 60.4% HIDES = 74.4% |

* Screening out tools

**Supplementary Table 5 Domains covered by tools used for Emergency Department attendees**

| **Lead Author (year of publication)** | **Number of questions** | **Demographics** | **Symptoms and signs** | **Indicator conditions** | **Risk behaviours** |
| --- | --- | --- | --- | --- | --- |
| Lyons (2013)^38^* | Over 50 items (See supplemental electronic appendix) |  | signs and symptoms of HIV |  | risk behaviours, homelessness, mental illness, STD exposure or infection, violence, substance use, pregnancy, incarceration |
| Dowdy (2011)^39^* | unclear |  | "HIV-related symptoms" |  | "HIV risk factors" |
| Hsieh (2018)^40^* | Denver HIV risk score compared with traditional targeting (CDC risk behaviours) and non-targeted |  |  |  |  |
| Hsieh (2014)^41^* | 7 (Uses modified abbreviated Denver HIV Risk Score) | Age, gender, race/ethnicity |  |  | Sex with male (MSM), IDU, past HIV testing |
| Haukoos (2015)^42^* | 8 (Uses Denver HIV risk score) | Age, gender, race/ethnicity |  |  | Sex with a male, injection drug use, past HIV test |
| Haukoos (2013)^43^* | 8 (uses Denver HIV risk score) | Age, gender, race/ethnicity |  |  | Sex with male, vaginal sex, receptive anal intercourse, IDU, past HIV test |
| Haukoos (2017)^44^* | 8 (Uses Denver HIV risk score) | Age, gender, race/ethnicity |  |  | Sex with male, vaginal sex, receptive anal intercourse, IDU, past HIV test |
| Gillet (2018)^45^* | 5 |  |  |  | MSM or IDU, condomless sex with MSM, IVDU, sex worker or partner with STI or known to be HIV+, condomless sex with partners from Caribbean, Eastern Europe or SE Asia or while travelling in these regions |
| Haukoos (2012)^46^* | 8 (uses Denver HIV risk score) | Age, gender, race/ethnicity |  |  | Sex with male, vaginal sex, receptive anal intercourse, IDU, past HIV test |
| Elias (2016)^47^ | 20 |  |  | STI, lymphoma, cancer, herpes zoster, mononucleosis-like syndrome, Hepatitis B or C, Thrombopenia, seborrheic dermatitis, oral candidiasis, oral hairy leukoplakia, unexplained fever, unexplained diarrhoea (>3 months), unexplained weight loss, *Mycobacterium tuberculosis* disease | Unprotected sexual intercourse, partner with HIV infection, man with man sex, hemoderivative transfusion, parental illicit or recreational drug use, any suspicion of HIV acquisition |
| Ayerbe (2016)^48^ | 20 |  |  | STI, lymphoma, cancer, herpes zoster, mononucleosis-like syndrome, Hepatitis B or C, Thrombopenia, seborrheic dermatitis, oral candidiasis, oral hairy leukoplakia, unexplained fever, unexplained diarrhoea (>3 months), unexplained weight loss, *Mycobacterium tuberculosis* disease | Unprotected sexual intercourse, partner with HIV infection, man with man sex, hemoderivative transfusion, parental illicit or recreational drug use, any suspicion of HIV acquisition |

* Screening out tools

**Supplementary Table 6 Risk-based screening tools for Primary care attendees**

| **Lead Author (year of publication)** | **Year of data** | **Sample size** | **Country** | **Setting** | **External validation?** | **AUC** | **Sensitivity** | **Specificity** |
| --- | --- | --- | --- | --- | --- | --- | --- | --- |
| Muttai (2020)^49^ | 2017-18 | 19458 (development) + 11330 (validation) | Kenya | health facilities (>=15, documentation of one or more behavioural risk characteristics) | 1 (development and validation datasets) | Development - AUC 0.69 (0.53-0.84); Validation - AUC 0.69 (0.6-0.77) |  |  |
| Menacho (2013)^50^ | 2009-11 | 85 (indicator strategy) + 304 (non-indicator strategy) | Spain | Primary care centres (18-65) | no |  |  |  |
| Krakower (2019)^51^ | 2007-15 | 1155966 (development cohort), 537 257 for prospective validation cohort, 33404 for external validation cohort | USA | ambulatory care (>=15) | 1. this study | AUC 0.86 (0.82-0.9) development cohort; AUC 0.91 (0.81-1) for prospective validation, 0.77 (0.74-0.79) for external validation |  |  |
| Joseph (2018)^52^ | 2017 | 88641 | Kenya | health facilities - outpatients and inpatients (>10) | no |  |  |  |
| Damery (2013)^53^ | 1989-2010 | 3515 | UK | GP (THIN database, >=18, diagnosed with HIV or AIDS at least 1 year before registering) | no | 0.66 | 42% | 80% |
| Manavi (2012)^54^ | unclear | 3515 | UK | GP | no |  |  |  |
| Aim-Eusebi (2018)^55^ | 2014 | 842 | France | GP (first 50 patients attending participating GPs, irrespective of the reason for consultation, 18-65) |  | no difference in the number of HIV tests before and after intervention |  |  |
| Cayuelas Redondo (2019)^56^ | 2014-15 | 832 | Spain | Primary health care (18-65, new diagnosis of predefined indicator condition) | unclear |  |  |  |
| Elias (2016)^47^ | 2012-2013 | 5329 | Spain | ED and primary care centre (18-60) | no |  | 100% | 49% |
| Ayerbe (2016)^48^ | 2016 | 5329 | Spain | ED and primary care centres | no | RE&CI-Q: Sens 100%, spec 49%, PPV 0.8%, NPV 100%. Denver HIV risk score: Sens 72.7%, spec 60.4%, PPV 0.76%, NPV 99.8%; HIV indicator diseases across Europe Study (HIDES): Sens 91%, spec 74.4%, PPV 1.4%, NPV 99.9% | RE&CI-Q = 100% Denver HIV risk score = 72.7% HIDES = 91% | RE&CI-Q = 49% Denver HIV risk score = 60.4% HIDES = 74.4% |

**Supplementary Table 7 Domains covered by tools used for Primary care attendees**

| **Lead Author (year of publication)** | **Number of questions** | **Demographics** | **Symptoms and signs** | **Indicator conditions** | **Risk behaviours** |
| --- | --- | --- | --- | --- | --- |
| Muttai (2020)^49^ | 10 | age 35-44, manual/domestic occupation, trade/sales/service occupation, married polygamous, widowed, separated, divorced |  |  | >=2 sexual partners in prior 12 months, STI Rx in prior 12 months, never tested for HIV, HIV neg result > 12 months ago |
| Menacho (2013)^50^ | 4 |  | Herpes zoster, seborrhoeic eczema, mononucleosis syndrome, leucopenia/thrombocytopenia |  |  |
| Krakower (2019)^51^ | 23 | Black race, White race, Male, Years of previous electronic health record (EHR) data, at least 1 year of previous EHR data, at least 2 years of previous EHR data, any data on primary language |  | Syphilis | HIV counselling in last 2 years, contact of STI, number of positive gonorrhoea test in last 2 years, number of chlamydia test, number of HIV test, number of HIV ELISA test, number of HIV test in the last 2 years, number of HIV RNA test in previous year, testing for acute, testing for acute HIV in last 2 years, prescribed benzathine penicillin, prescribed benzathine penicillin in the previous year, prescribed benzathine penicillin in last 2 years, prescribed buprenorphine and naloxone in last 2 years |
| Joseph (2018)^52^ | 5 |  |  |  | Never tested, last negative HIV test 12 or more months prior or unknown date of last test, reporting recent HIV exposure or signs or symptoms of TB or a sexually transmitted infection (STI) |
| Damery (2013)^53^ | 37 |  |  | 37 clinical indicators (Box 1) |  |
| Manavi (2012)^54^ | 12 |  |  | Bacterial pneumonia, oral candidiasis, herpes zoster, non-Hodgkin's lymphoma, lymphadenopathy, STI, fever of unknown origin, blood dyscrasia, diarrhoea- two consultations, diarrhoea- one consultation, weight loss | Living in deprived areas |
| Aim-Eusebi (2018)^55^ | 7 | Born in an endemic country |  |  | Tattoos and/or piercings, multiple partners in last 12 months, history of STIs, MSM, Hx of blood transfusion before 1992, IDU |
| Cayuelas Redondo (2019)^56^ | 11 |  |  | Herpes zoster, Mononucleosis syndrome, leucopenia/thrombocytopenia >4 weeks, seborrheic eczema, STI (genital herpes, urethritis, cervicitis, gonorrhoea, genital ulcer, syphilis, LGV, condyloma, vaginosis), cervical dysplasia, acute/chron HBV/HCV, idiopathic lymphadenopathy, diarrhoea>4 weeks, unexplained weight loss, community-acquired pneumonia |  |
| Elias (2016)^47^ | 20 |  |  | STI, lymphoma, cancer, herpes zoster, mononucleosis-like syndrome, Hepatitis B or C, Thrombopenia, seborrheic dermatitis, oral candidiasis, oral hairy leukoplakia, unexplained fever, unexplained diarrhoea (>3 months), unexplained weight loss, *Mycobacterium tuberculosis* disease | Unprotected sexual intercourse, partner with HIV infection, man with man sex, hemoderivative transfusion, parental illicit or recreational drug use, any suspicion of HIV acquisition |
| Ayerbe (2016)^48^ | 20 |  |  | STI, lymphoma, cancer, herpes zoster, mononucleosis-like syndrome, Hepatitis B or C, Thrombopenia, seborrheic dermatitis, oral candidiasis, oral hairy leukoplakia, unexplained fever, unexplained diarrhoea (>3 months), unexplained weight loss, *Mycobacterium tuberculosis* disease | Unprotected sexual intercourse, partner with HIV infection, man with man sex, hemoderivative transfusion, parental illicit or recreational drug use, any suspicion of HIVacquisitionn |

**Supplementary Table 8 Risk-based screening tools for Hospital inpatients**

| **Lead Author (year of publication)** | **Year of data** | **Sample size** | **Country** | **Setting (target group)** | **External validation?** | **AUC** | **Sensitivity** | **Specificity** |
| --- | --- | --- | --- | --- | --- | --- | --- | --- |
| Allison (2011)**^18^** | 2007-08 | 487 | PNG | Hospital (Children admitted to paediatric ward) | Yes - study population split into sample set and validation set |  | 96.3-29.1% | 25.0-92.6% |
| Allison (2010)**^19^** | 2007-08 | 487 | PNG | Hospital inpatients (Pediatric patients at PMGH, 1-80 months) | unclear |  | 96% |  |
| Felsen (2017)**^57^** | 2013-15 | 55553 | USA | Hospital (age 21-64 admitted to hospital) | no |  |  |  |
| Leal (2016)**^58^** | 2003-13 | 138 | Spain | Tertiary hospital (hospital attendees) | no | AUC 0.78 (0.69-0.84) | 64% | 74% |
| Nathoo (2012)^29^ | 2012 | 355 | Zimbabwe | Medical paediatric wards (hospitalised children aged 2 to 18 months) | no |  | 43-49% | 72.3-89.5% |

**Supplementary Table 9 Domains covered by tools used for Hospital inpatients**

| **Lead Author (year of publication)** | **Number of questions** | **Demographics** | **Symptoms and signs** | **Indicator conditions** | **Risbehavioursrs** |
| --- | --- | --- | --- | --- | --- |
| Allison (2011)**^18^** | 4 |  | low weight for age, persistent fever (for >=1 month), enlarged lymph nodes (neck, axillae, groin), oral candidiasis |  |  |
| Allison (2010)**^19^** | 4 |  | Persistent fever, lymphadenopathy, underweight for age, oral candidiasis |  |  |
| Felsen (2017)**^57^** | 2 |  |  |  | No HIV test in the electronic medical record or had a high-risk diagnosis after last document HIV test. (High risk includes STI, Hepatitis B/C, substance use, HIV indicator conditions or AIDS-related conditions as per ICD9-CM) |
| Leal (2016)**^58^** | 4 |  |  |  | MSM, known HIV+ partner, previous post-exposure prophylaxis, previous STI |
| Nathoo (2012)^29^ | 20 |  | Presenting symptoms: Parent with tuberculosis, diarrhoea, vomiting, rash, ear discharge, parotid swelling, weight loss  Clinical features: pallor, cyanosis, pneumonia, jaundice, oedema, generalised lymphadenopathy, clubbing, rash, parotid swelling, oral thrush, ear discharge, hepatomegaly, splenomegaly |  |  |

**Supplementary Table 10 Risk-based screening tools for Adults in the community**

| **Lead Author (year of publication)** | **Year of data** | **Sample size** | **Country** | **Setting (target group)** | **External validation?** | **AUC** | **Sensitivity** | **Specificity** |
| --- | --- | --- | --- | --- | --- | --- | --- | --- |
| Ayieko**^59^** | 2017 | 4159 | Kenya | Rural community, adults aged >15 years | Unclear |  |  |  |
| Giovenco**^60^** | 2011-2015 | 2178 | South Africa | School attending, 13-20 year olds, not married/pregnant | Yes | 0.55 (0.44-0.65) | 85% | 6% |

**Supplementary Table 11 Domains covered by tools used for Adults in the community**

| **Lead Author (year of publication)** | **Number of questions** | **Demographics** | **Symptoms and signs** | **Indicator conditions** | **Risk behaviours** |
| --- | --- | --- | --- | --- | --- |
| Ayieko**^59^** | 8 | age, sex, marital status, education, circumcision, occupation |  |  | polygamy, alcohol use |
| Giovenco**^60^** | 6 | age, living with primary partner, partner provides financial/material support |  |  | partner with other partners, alcohol use in last 3 months, HSV2 serostatus |

**Supplementary Table 12 Risk-based screening tools for STI clinic attendees**

| **Lead Author (year of publication)** | **Year of data** | **Sample size** | **Country** | **Setting (target group)** | **External validation?** | **AUC** | **Sensitivity** | **Specificity** |
| --- | --- | --- | --- | --- | --- | --- | --- | --- |
| Facente (2011)^61^ | 2004-07 | 12622 | USA | STI clinic (STI clinic attendees) | No | 0.669 | 83.3% (67.2-93.6) | 50.4% (49.5-51.3) |
| Falasinnu (2015)^62^ | 2000-12 | 47175 | Canada | STI clinic (STI clinic attendees) | Yes | AUC 0.80 (0.79-0.81) | 96% |  |
| Sanders (2015)^7^ | 1993-98 (Mombassa), 2005-12 (Kilifi) | 122 AHI + 45,961 uninfected | Kenya, Malawi, South Africa | STI clinic (FSW, MSM, STI clinic attendees) | Yes | AUC 0.78 (0.61-0.89) | 90% in Kilifi, 93% in Lilongwe | 85% in Mombasa, 74% in Kilifi |

**Supplementary Table 13 Domains covered by tools used for STI clinic attendees**

| **Lead Author (year of publication)** | **Number of questions** | **Demographics** | **Symptoms and signs** | **Indicator conditions** | **Risk behaviours** |
| --- | --- | --- | --- | --- | --- |
| Facente (2011)^61^ | 5 |  |  |  | MSM, IDU, HIV-positive partner, condomless receptive anal sex, STI |
| Falasinnu (2015)^62^ | 8 | age, gender, race/ethnicity |  |  | sex with male, vaginal sex, receptive anal sex, IDU, past HIV test |
| Sanders (2015)^7^ | 7 | age | fever, diarrhoea, fatigue, body pains, sore throat, genital ulcer |  |  |

**Supplementary Table 14 Risk-based screening tools for incarcerated persons**

| **Lead Author (year of publication)** | **Year of data** | **Sample size** | **Country** | **Setting (target group)** | **External validation?** | **AUC** | **Sensitivity** | **Specificity** |
| --- | --- | --- | --- | --- | --- | --- | --- | --- |
| Nelwan (2016)^63^ | 2007-10 | 662 (routine), 888 (targeted) | Indonesia | Prison (Male prisoners convicted for drug-related offences) | No |  |  |  |
| Sampson (2011)^64^ | 2002-05 | 3610 | USA | Prison (Inmates) | No |  | 82.6% (71.2-94) |  |

**Supplementary Table 15 Domains covered by tools used for incarcerated persons**

| **Lead Author (year of publication)** | **Number of questions** | **Demographics** | **Symptoms and signs** | **Indicator conditions** | **Risbehavioursrs** |
| --- | --- | --- | --- | --- | --- |
| Nelwan (2016)^63^ | 1 |  |  |  | Asked if ever injected drugs and examined for needle track changes or other signs of IDU |
| Sampson (2011)^64^ | 3 | age, ever tested for HIV, race/ethnicity |  |  |  |

**Supplementary Table 16 Risk-based screening tools for serodiscordant couples**

| **Lead Author (year of publication)** | **Year of data** | **Sample size** | **Country** | **Setting** | **External validation?** | **AUC** | **Sensitivity** | **Specificity** |
| --- | --- | --- | --- | --- | --- | --- | --- | --- |
| Kahle (1999)^65^ | 2004-10 | 8651 | Botswana, Kenya, Rwanda, South Africa, Tanzania, Uganda, Zambia | unclear | Yes | AUC 0.74 (0.7-0.78), External validation AUC 0.76 (0.7-0.83) |  |  |
| Okpokoro (2016)^66^ | unclear | 500 | Nigeria | unclear | Yes |  |  |  |

**Supplementary Table 17 Domains covered by tools used for serodiscordant couples**

| **Lead Author (year of publication)** | **Number of questions** | **Demographics** | **Symptoms and signs** | **Indicator conditions** | **Risk behaviours** |
| --- | --- | --- | --- | --- | --- |
| Kahle (1999)^65^ | 6 | age of HIV uninfected partner, married and/or cohabiting partnership, number of children, uncircumcised male HIV uninfected partner, plasma HIV RNA in HIV infected partner |  |  | Unprotected sex |
| Okpokoro (2016)^66^ | 4 | Age of the HIV-1 uninfected partner, married and/or cohabiting partnership, and plasma HIV-1 RNA in the HIV-1 infected partner |  |  | Unprotected sex |

**Supplementary Table 18 Risk-based screening tools for people who inject drugs**

| **Lead Author (year of publication)** | **Year of data** | **Sample size** | **Country** | **Setting (target group)** | **External validation?** | **AUC** | **Sensitivity** | **Specificity** |
| --- | --- | --- | --- | --- | --- | --- | --- | --- |
| Smith (2015)^67^ | 1998-2008 | 1904 | USA | People who inject drugs | Yes | 0.72 | 86.2% | 42.5% |

**Supplementary Table 19 Domains covered by tools used for people who inject drugs**

| **Lead Author (year of publication)** | **Number of questions** | **Demographics** | **Symptoms and signs** | **Indicator conditions** | **Risk behaviours** |
| --- | --- | --- | --- | --- | --- |
| Smith (2015)^67^ | 7 | Age |  |  | Engagement in a methadone program, composite injection score (injection of heroin, injection of cocaine, sharing a cooker, sharing needles, visiting a shooting gallery) |

**Supplementary Table 20 Risk of bias assessment using Johanna Brigg's critical appraisal tool for cross-sectional studies** ^1^

| **Study** | **Were the criteria for inclusion in the sample clearly defined?** | **Were the study subjects and the setting described in detail?** | **Was the exposure measured in a valid and reliable way?** | **Were objective, standard criteria used for measurement of the condition?** | **Were confounding factors identified?** | **Were strategies to deal with confounding factors stated?** | **Were the outcomes measured in a valid and reliable way?** | **Was appropriate statistical analysis used?** |
| --- | --- | --- | --- | --- | --- | --- | --- | --- |
| Abbas^17^ | Yes | Yes | Yes | Yes | No | No | Yes | Unclear |
| Allison^18^ | Yes | Yes | Yes | Yes | Yes | Yes | Yes | Yes |
| Allison^19^ | Unclear | Yes | Yes | Yes | Yes | Yes | Yes | Yes |
| Bandason^21^ | Yes | Yes | Yes | Yes | Yes | Yes | Yes | Yes |
| Bandason^20^ | Yes | Yes | Yes | Yes | Yes | Unclear | Yes | Yes |
| Bandason^22^ | Yes | Yes | Yes | Unclear | Unclear | Unclear | Yes | Yes |
| Du Plessis^23^ | Yes | Yes | Yes | Yes | Yes | Yes | Yes | Yes |
| Elias^47^ | Yes | Yes | Yes | Yes | Yes | Yes | Yes | Yes |
| Facente^61^ | Yes | Yes | Yes | Yes | Yes | Yes | Yes | Yes |
| Falsinnu^62^ | No | No | Yes | Yes | No | No | Yes | Yes |
| Ferrand^24^ | Yes | Yes | Yes | Yes | Yes | Yes | Yes | Yes |
| Haukoos^42^ | Yes | Yes | Yes | Yes | Unclear | Unclear | Yes | Yes |
| Haukoos^68^ | Yes | Yes | Yes | Yes | Yes | Yes | Yes | Yes |
| Hoenigl^12^ | Yes | Yes | Yes | Yes | Yes | Yes | Yes | Yes |
| Hsieh^40^ | Yes | Yes | Yes | Yes | Unclear | Unclear | Yes | No |
| Hsieh^41^ | Unclear | Unclear | Yes | Yes | Unclear | Unclear | Yes | Yes |
| Ibrahim^25^ | Yes | Yes | Yes | Yes | N | N | Yes | N |
| Joseph^52^ | Yes | Yes | Yes | Yes | Yes | Yes | Yes | Yes |
| Kakanfo^26^ | No | No | Unclear | Unclear | Yes | Yes | Unclear | Yes |
| Lin^3^ | Yes | Yes | Yes | Yes | Yes | Yes | Yes | Yes |
| Mafaune^27^ | Unclear | Yes | Yes | Yes | Yes | Unclear | Yes | Yes |
| Menacho^50^ | Yes | Yes | Yes | Yes | Unclear | Unclear | Yes | Yes |
| Moucheraud^28^ | Yes | Yes | Yes | Yes | Yes | Yes | Yes | Yes |
| Nathoo^29^ | Yes | Yes | Yes | Yes | Yes | Yes | Yes | Yes |
| Nelwan^63^ | Yes | Yes | Yes | Yes | Yes | Yes | Yes | Yes |
| Sampson^64^ | Yes | No | Yes | Yes | No | Not applicable | Yes | Yes |
| Sanders^7^ | Yes | Yes | Yes | Yes | Unclear | Unclear | Yes | Yes |
| Sibanda^69^ | Yes | Yes | Yes | Yes | Unclear | Unclear | Yes | Yes |
| Yin^10^ | Yes | Yes | Not applicable | Yes | Yes | Unclear | Yes | Yes |
| Yumo^70^ | Yes | Yes | Yes | Yes | No | Not applicable | Yes | Yes |

**Supplementary Table 21 Risk of bias assessment using Johanna Brigg's critical appraisal tool for case-control studies** ^1^

|  | **Were the groups comparable other than the presence of disease in cases or the absence of disease in controls?** | **Were cases and controls matched appropriately?** | **Were the same criteria used for identification of cases and controls?** | **Was exposure measured in a standard, valid and reliable way?** | **Was exposure measured in the same way for cases and controls?** | **Were confounding factors identified?** | **Were strategies to deal with confounding factors stated?** | **Were outcomes assessed in a standard, valid and reliable way for cases and controls?** | **Was the exposure period of interest long enough to be meaningful?** | **Was appropriate statistical analysis used?** |
| --- | --- | --- | --- | --- | --- | --- | --- | --- | --- | --- |
| Caldwell^71^ | Yes | Yes | Yes | Yes | Yes | Yes | Yes | Yes | N/A | Yes |
| Cayuelas^56^ | Yes | Yes | Yes | Yes | No | Yes | Yes | Unclear | Yes | Yes |
| Damery^53^ | Yes | Yes | Yes | Unclear | Unclear | Yes | Yes | Yes | Yes | Yes |
| Leal^58^ | Yes | Yes | Yes | Yes | Yes | Yes | Yes | Yes | Yes | Yes |
| Lin^2^ | Yes | Yes | Yes | Yes | Yes | Yes | Yes | Yes | Yes | Yes |
| Manavi^54^ | Yes | Yes | Yes | Yes | Yes | Unclear | Unclear | Yes | Unclear | Yes |

**Supplementary Table 22 Risk of bias assessment using Johanna Brigg's critical appraisal tool for cohort studies** ^1^

|  | **Were the two groups similar and recruited from the same population?** | **Were the exposures measured similarly to assign people to both exposed and unexposed groups?** | **Was the exposure measured in a valid and reliable way?** | **Were confounding factors identified?** | **Were strategies to deal with confounding factors stated?** | **Were the groups/participants free of the outcome at the start of the study (or at the moment of exposure)?** | **Were the outcomes measured in a valid and reliable way?** | **Was the follow up time reported and sufficient to be long enough for outcomes to occur?** | **Was follow up complete, and if not, were the reasons to loss to follow up described and explored?** | **Were strategies to address incomplete follow up utilised?** | **Was appropriate statistical analysis used?** |
| --- | --- | --- | --- | --- | --- | --- | --- | --- | --- | --- | --- |
| **Aim-Eusebi^55^** | Yes | Yes | Unclear | Yes | Unclear | N/A | N/A | Yes | Yes | N/A | Yes |
| **Beymer^16^** | Yes | Yes | Yes | Yes | No | Unclear | Yes | Yes | Yes | N/A | Yes |
| **Burgess^32^** | Yes | Yes | Yes | Yes | No | Unclear | Yes | Yes | Unclear | Unclear | Yes |
| **Dijkstra^4^** | Yes | Yes | Yes | Yes | Yes | Unclear | Unclear | Yes | Yes | Unclear | Yes |
| **Dijkstra^11^** | Yes | Yes | Unclear | Yes | Yes | Unclear | Unclear | Yes | Yes | Unclear | Yes |
| **Jones^14^** | Yes | Yes | Yes | Yes | Yes | Yes | Yes | Yes | N/A | N/A | Yes |
| **Kahle^65^** | Yes | Yes | Yes | Yes | Yes | Yes | Yes | Yes | Yes | unclear | Yes |
| **Krakower^51^** | Yes | Yes | Yes | Yes | Yes | Yes | Yes | Yes | N/A | N/A | Yes |
| **Luo^13^** | Yes | Yes | Yes | Yes | Yes | Yes | Yes | Yes | Yes | Yes | Yes |
| **Muttai^49^** | Yes | Yes | Yes | Unclear | Unclear | Yes | Yes | Unclear | Unclear | Unclear | Yes |
| **Peebles^34^** | Yes | Yes | Yes | Yes | Yes | Yes | Yes | Yes | Yes | Unclear | Yes |
| **Pintye^35^** | Yes | Yes | Yes | Yes | Yes | Yes | Yes | Yes | Unclear | Unclear | Yes |
| **Sanders (Mombasa)^7^** | Yes | Yes | Yes | Unclear | Unclear | Yes | Yes | Yes | Unclear | Unclear | Yes |
| **Sanders (Kilifi)^7^** | Yes | Yes | Yes | Unclear | Unclear | Yes | Yes | Yes | Unclear | Unclear | Yes |
| **Sanders (Durban)^7^** | Yes | Yes | Yes | Unclear | Unclear | Yes | Yes | Yes | Unclear | Unclear | Yes |
| **Smith^67^** | Yes | Yes | Yes | Yes | Unclear | Yes | Yes | Unclear | Unclear | Unclear | Yes |
| **Wahome^72^** | Yes | Unclear | Yes | Yes | Yes | Yes | Yes | Yes | No | No | Yes |
| **Wahome^6^** | Yes | Yes | Yes | Yes | Unclear | Yes | Yes | Yes | Unclear | Unclear | Yes |
| **Yun^15^** | Yes | Yes | Yes | Yes | Yes | Yes | Yes | Yes | Yes | Unclear | Yes |

**Supplementary Table 23 Risk of bias assessment using Johanna Brigg's critical appraisal tool for quasi-experimental studies** ^1^

|  | **Is it clear in the study what is the 'cause' and what is the 'effect' (i.e. there is no confusion about which variable comes first)?** | **Were the participants included in any comparisons similar?** | **Were the participants included in any comparisons receiving similar treatment/care, other than the exposure or intervention of interest?** | **Was there a control group?** | **Were there multiple measurements of the outcome both pre and post the intervention/exposure?** | **Was follow up complete and if not, were differences between groups in terms of their follow up adequately described and analysed?** | **Were the outcomes of participants included in any comparisons measured in the same way?** | **Were outcomes measured in a reliable way?** | **Was appropriate statistical analysis used?** |
| --- | --- | --- | --- | --- | --- | --- | --- | --- | --- |
| **Felsen^73^** | Yes | Yes | Yes | Yes | Yes | Yes | Yes | Yes | Yes |
| **Haukoos^43^** | Yes | Yes | Yes | Yes | Yes | Yes | Yes | Yes | Yes |
| **Yumo^74^** | Yes | Yes | Yes | No | Yes | Unclear | Yes | Yes | Yes |

**Supplementary Table 24 Risk of bias assessment using Johanna Brigg's critical appraisal tool for randomised controlled studies** ^1^

|  | **Was true randomisation used for assignment of participants to treatment groups?** | **Was allocation to treatment groups concealed?** | **Were treatment groups similar at the baseline?** | **Were participants blind to treatment assignment?** | **Were those delivering treatment blind to treatment assignment?** | **Were outcomes assessors blind to treatment assignment?** | **Were treatment groups treated identically other than the intervention of interest?** | **Was follow up complete and if not, were differences between groups in terms of their follow up adequately described and analysed?** | **Were participants analysed in the groups to which they were randomised?** | **Were outcomes measured in the same way for treatment groups?** | **Were outcomes measured in a reliable way?** | **Was appropriate statistical analysis used?** | **Was the trial design appropriate, and any deviations from the standard RCT design (individual randomisation, parallel groups) accounted for in the conduct and analysis of the trial?** |
| --- | --- | --- | --- | --- | --- | --- | --- | --- | --- | --- | --- | --- | --- |
| **Ayieko^59^** | Unclear | Unclear | Yes | Unclear | Unclear | Unclear | Unclear | N/A | Unclear | Yes | Yes | Yes | N/A |
| **Balkus (VOICE)^30^** | Yes | Unclear | Yes | Yes | Unclear | Unclear | Yes | Yes | Yes | Yes | Yes | Yes | Yes |
| **Balkus (HPTN035)^30^** | Yes | No | Yes | Yes | Yes | Yes | Yes | Yes | Yes | Yes | Yes | Yes | Yes |
| **Balkus (FEM-PrEP)^30^** | Yes | Unclear | Yes | Yes | Unclear | Yes | Yes | Yes | Unclear | Yes | Yes | Yes | Yes |
| **Balkus^31^** | Yes | Yes | Yes | Yes | Yes | Yes | Yes | Yes | Yes | Yes | Yes | Yes | Yes |
| **Blumenthal^75^** | Yes | Unclear | Yes | No | Unclear | Unclear | Yes | Yes | Yes | Yes | Yes | Yes | Yes |
| **Burgess^33^** | Yes | Yes | Yes | Yes | Yes | Yes | Yes | Yes | Yes | Yes | Yes | Yes | Yes |
| **Gillet^45^** | Yes | Yes | Yes | Unclear | Yes | Yes | Yes | Yes | Yes | Yes | Yes | Yes | Yes |
| **Giovenco^60^** | Yes | Yes | Yes | Unclear | Yes | Yes | Yes | Yes | Yes | Yes | Yes | Yes | Yes |
| **Haukoos^44^** | Yes | Yes | Unclear | Unclear | Unclear | Unclear | Unclear | Unclear | Yes | Yes | Yes | Yes | Yes |
| **Kahle (RCT1)^65^** | Yes | Unclear | Yes | Yes | Yes | Yes | Yes | Yes | Unclear | Yes | Yes | Yes | Yes |
| **Kahle (RCT2)^65^** | Y | Yes | Yes | Unclear | Unclear | Unclear | Unclear | Unclear | Unclear | Yes | Yes | Yes | Yes |
| **Lyons^38^** | Y | Unclear | Yes | No | No | Unclear | Yes | Yes | No | Yes | Yes | Yes | Yes |
| **Scott^5^** | Yes | Yes | Yes | Yes | Yes | Yes | Yes | Yes | Yes | Yes | Yes | Yes | Unclear |
| **Smith^9^** | Yes | Yes | Yes | Yes | Yes | Yes | Yes | Yes | Yes | Yes | Yes | Yes | Yes |
| **Wand^36^** | Yes | Yes | Yes | Yes | Yes | No | Yes | Yes | Yes | Yes | Yes | Yes | Yes |
| **Wand^37^** | Yes | Yes | Yes | Yes | Yes | No | Yes | Yes | Yes | Yes | Yes | Yes | Yes |

**REFERENCES**

1. Joanna Briggs Institute Critical Appraisal Tools. <https://joannabriggs.org/critical-appraisal-tools2020>).

2. Lin TC, Dijkstra M, De Bree GJ, Schim Van Der Loeff MF, Hoenigl M. The Amsterdam symptom and risk-based score predicts for acute HIV infection in men who have sex with men in San Diego. *Journal of Acquired Immune Deficiency Syndromes* 2018; **79**(2): E52-E5.

3. Lin TC, Gianella S, Tenenbaum T, Little SJ, Hoenigl M. A Simple Symptom Score for Acute Human Immunodeficiency Virus Infection in a San Diego Community-Based Screening Program. *Clinical infectious diseases : an official publication of the Infectious Diseases Society of America* 2018; **67**(1): 105-11.

4. Dijkstra M, de Bree GJ, Stolte IG, et al. Development and validation of a risk score to assist screening for acute HIV-1 infection among men who have sex with men (vol 17, pg 425, 2017). *Bmc Infectious Diseases* 2017; **17**.

5. Scott H, Vittinghoff E, Irvin R, et al. Development and Validation of the Personalized Sexual Health Promotion (SexPro) HIV Risk Prediction Model for Men Who Have Sex with Men in the United States. *AIDS and behavior* 2020; **24**(1): 274-83.

6. Wahome E, Fegan G, Okuku HS, et al. Evaluation of an empiric risk screening score to identify acute and early HIV-1 infection among MSM in Coastal Kenya. *AIDS (London, England)* 2013; **27**(13): 2163-6.

7. Sanders EJ, Wahome E, Powers KA, et al. Targeted screening of at-risk adults for acute HIV-1 infection in sub-Saharan Africa. *AIDS* 2015; **29 Suppl 3**: S221-30.

8. Wahome E, Thiong'o AN, Mwashigadi G, et al. An Empiric Risk Score to Guide PrEP Targeting Among MSM in Coastal Kenya. *AIDS and behavior* 2018; **22**(Suppl 1): 35-44.

9. Smith DK, Pals SL, Herbst JH, Shinde S, Carey JW. Development of a clinical screening index predictive of incident HIV infection among men who have sex with men in the United States. *Journal of acquired immune deficiency syndromes (1999)* 2012; **60**(4): 421-7.

10. Yin L, Zhao Y, Peratikos MB, et al. Risk Prediction Score for HIV Infection: Development and Internal Validation with Cross-Sectional Data from Men Who Have Sex with Men in China. *AIDS and behavior* 2018; **22**(7): 2267-76.

11. Dijkstra M, Lin TC, de Bree GJ, Hoenigl M, Schim van der Loeff MF. Validation of the San Diego Early Test Score for Early Human Immunodeficiency Virus Infection Among Amsterdam Men Who Have Sex With Men. *Clinical infectious diseases : an official publication of the Infectious Diseases Society of America* 2020; **70**(10): 2228-30.

12. Hoenigl M, Graff-Zivin J, Little SJ. Costs per Diagnosis of Acute HIV Infection in Community-based Screening Strategies: A Comparative Analysis of Four Screening Algorithms. *Clinical Infectious Diseases* 2015; **62**(4): 501-11.

13. Luo Q, Huang X, Li L, et al. External validation of a prediction tool to estimate the risk of human immunodeficiency virus infection amongst men who have sex with men. *Medicine* 2019; **98**(29): e16375.

14. Jones J, Hoenigl M, Siegler AJ, Sullivan PS, Little S, Rosenberg E. Assessing the Performance of 3 Human Immunodeficiency Virus Incidence Risk Scores in a Cohort of Black and White Men Who Have Sex With Men in the South. *Sex Transm Dis* 2017; **44**(5): 297-302.

15. Yun K, Xu J, Leuba S, et al. Development and Validation of a Personalized Social Media Platform-Based HIV Incidence Risk Assessment Tool for Men Who Have Sex With Men in China. *J Med Internet Res* 2019; **21**(6): e13475.

16. Beymer MR, Weiss RE, Sugar CA, et al. Are Centers for Disease Control and Prevention Guidelines for Preexposure Prophylaxis Specific Enough? Formulation of a Personalized HIV Risk Score for Pre-Exposure Prophylaxis Initiation. *Sex Transm Dis* 2017; **44**(1): 48-56.

17. Abbas AA, Gabo NEAAA, Babiker ZOE, Herieka EAM. Paediatric HIV in central Sudan: high sero-prevalence and poor performance of clinical case definitions. *Journal of clinical virology : the official publication of the Pan American Society for Clinical Virology* 2010; **47**(1): 82-4.

18. Allison WE, Kiromat M, Vince J, et al. Development of a clinical algorithm to prioritise HIV testing of hospitalised paediatric patients in a low resource moderate prevalence setting. *Arch Dis Child* 2011; **96**(1): 67-72.

19. Allison W, Kiromat M, Vince J, Wand H, Cunningham P, Kaldor J. Development of an algorithmic tool to guide routine HIV testing of hospitalized pediatric patients in Papua New Guinea (PNG), a resource-limited setting with moderate HIV prevalence. *Journal of the International Association of Physicians in AIDS Care* 2010; **9**(1): 56.

20. Bandason T, McHugh G, Dauya E, et al. Validation of a screening tool to identify older children living with HIV in primary care facilities in high HIV prevalence settings. *AIDS (London, England)* 2016; **30**(5): 779-85.

21. Bandason T, Dauya E, Dakshina S, et al. Screening tool to identify adolescents living with HIV in a community setting in Zimbabwe: A validation study. *PloS one* 2018; **13**(10): e0204891.

22. Bandason T, McHugh G, Ferrand RA, Munyati S, Kranzer K, Chonzi P. Moving towards targeted HIV testing in older children at risk of vertically transmitted HIV. *Journal of the International AIDS Society* 2015; **18**: 82.

23. Du Plessis NM, Muller CJB, Avenant T, Pepper MS, Goga AE. An Early Infant HIV Risk Score for Targeted HIV Testing at Birth. *Pediatrics* 2019; **143**(6).

24. Ferrand RA, Weiss HA, Nathoo K, et al. A primary care level algorithm for identifying HIV-infected adolescents in populations at high risk through mother-to-child transmission. *Trop Med Int Health* 2011; **16**(3): 349-55.

25. Ibrahim M, Maswabi K, Ajibola G, et al. Targeted HIV testing at birth supported by low and predictable mother-to-child transmission risk in Botswana. *Journal of the International AIDS Society* 2018; **21**(5): e25111.

26. Kakanfo K, Khamofu H, Obiora-Okafo C, et al. How well does the bandason HIV risk screening tool perform in the nigerian setting? findings from a large-scale field implementation. *AIDS Research and Human Retroviruses* 2018; **34**(Supplement 1): 151.

27. Mafaune HW, Sacks E, Chadambuka A, et al. Effectiveness of Maternal Transmission Risk Stratification in Identification of Infants for HIV Birth Testing: Lessons From Zimbabwe. *Journal of acquired immune deficiency syndromes (1999)* 2020; **84 Suppl 1**(100892005): S28-S33.

28. Moucheraud C, Chasweka D, Nyirenda M, Schooley A, Dovel K, Hoffman RM. Simple Screening Tool to Help Identify High-Risk Children for Targeted HIV Testing in Malawian Inpatient Wards. *Journal of acquired immune deficiency syndromes (1999)* 2018; **79**(3): 352-7.

29. Nathoo KJ, Rusakaniko S, Tobaiwa O, Mujuru HA, Ticklay I, Zijenah L. Clinical predictors of HIV infection in hospitalized children aged 2-18 months in Harare, Zimbabwe. *Afr Health Sci* 2012; **12**(3): 259-67.

30. Balkus JE, Brown E, Palanee T, et al. An Empiric HIV Risk Scoring Tool to Predict HIV-1 Acquisition in African Women. *Journal of acquired immune deficiency syndromes (1999)* 2016; **72**(3): 333-43.

31. Balkus J, Palnee-Phillips T, Zhang J, et al. A Validated Risk Score to Predict HIV Acquisition in African Women: Assessing Risk Score Performance among Women who Participated in the ASPIRE Trial. HIV Research for Prevention (HIVR4P) Partnering for Prevention; 2016; Chicago; 2016.

32. Burgess EK, Yende-Zuma N, Castor D, Karim QA. An age-stratified risk score to predict HIV acquisition in young South African women. *Topics in Antiviral Medicine* 2018; **26**: 419.

33. Burgess EK, Delany-Moretlwe S, Pisa P, et al. Validation of a risk score for HIV acquisition in young African women with facts 001. *Topics in Antiviral Medicine* 2017; **25**(1 Supplement 1): 364s-5s.

34. Peebles K, Palanee-Phillips T, Balkus JE, et al. Age-Specific Risk Scores Do Not Improve HIV-1 Prediction Among Women in South Africa. *J Acquir Immune Defic Syndr* 2020; **85**(2): 156-64.

35. Pintye J, Drake AL, Kinuthia J, et al. A Risk Assessment Tool for Identifying Pregnant and Postpartum Women Who May Benefit From Preexposure Prophylaxis. *Clinical infectious diseases : an official publication of the Infectious Diseases Society of America* 2017; **64**(6): 751-8.

36. Wand H, Ramjee G. Assessing and evaluating the combined impact of behavioural and biological risk factors for HIV seroconversion in a cohort of South African women. *AIDS care* 2012; **24**(9): 1155-62.

37. Wand H, Reddy T, Naidoo S, et al. A Simple Risk Prediction Algorithm for HIV Transmission: Results from HIV Prevention Trials in KwaZulu Natal, South Africa (2002-2012). *AIDS Behav* 2018; **22**(1): 325-36.

38. Lyons MS, Lindsell CJ, Ruffner AH, et al. Randomized comparison of universal and targeted HIV screening in the emergency department. *J Acquir Immune Defic Syndr* 2013; **64**(3): 315-23.

39. Dowdy DW, Rodriguez RM, Hare CB, Kaplan B. Cost-effectiveness of targeted human immunodeficiency virus screening in an urban emergency department. *Academic emergency medicine : official journal of the Society for Academic Emergency Medicine* 2011; **18**(7): 745-53.

40. Hsieh Y, Patel A, Laeyendecker O, et al. All Current Emergency Department Screening Strategies for Human Immunodeficiency Virus Still Leaving Many Patients Undiagnosed. *Academic Emergency Medicine* 2018; **25**: S143-S4.

41. Hsieh YH, Haukoos JS, Rothman RE. Validation of an abbreviated version of the Denver HIV risk score for prediction of HIV infection in an urban ED. *Am J Emerg Med* 2014; **32**(7): 775-9.

42. Haukoos JS, Hopkins E, Bucossi MM, et al. Validation of a Quantitative HIV Risk Prediction Tool Using a National HIV Testing Cohort. *Jaids-Journal of Acquired Immune Deficiency Syndromes* 2015; **68**(5): 599-603.

43. Haukoos JS, Hopkins E, Bender B, et al. Comparison of enhanced targeted rapid HIV screening using the Denver HIV risk score to nontargeted rapid HIV screening in the emergency department. *Annals of emergency medicine* 2013; **61**(3): 353-61.

44. Haukoos J, Lyons M, White D, et al. A multi-center pragmatic randomized comparison of hiv screening strategy effectiveness in the emergency department: The hiv tested trial. *Annals of Epidemiology* 2017; **27**(8): 521.

45. Gillet C, Darling KEA, Senn N, Cavassini M, Hugli O. Targeted versus non-targeted HIV testing offered via electronic questionnaire in a Swiss emergency department: A randomized controlled study. *PloS one* 2018; **13**(3): e0190767.

46. Haukoos J, Hopkins E, Bender B, Sasson C, Al-Tayyib A, Thrun M. Enhanced targeted hiv screening using the denver hiv risk score outperforms nontargeted screening in the emergency department. *Academic Emergency Medicine* 2012; **19**(SUPPL. 1): S222-S3.

47. Elias MJP, Gomez-Ayerbe C, Elias PP, et al. Development and Validation of an HIV Risk Exposure and Indicator Conditions Questionnaire to Support Targeted HIV Screening. *Medicine* 2016; **95**(5): e2612.

48. Ayerbe CG, Muriel A, Reverte C, et al. Targeting HIV testing at a population level: cost effectiveness of three approaches. *Journal of the International AIDS Society* 2016; **19**: 230-2.

49. Muttai H, Guyah B, Musingila P, et al. Development and Validation of a Sociodemographic and Behavioral Characteristics-Based Risk-Score Algorithm for Targeting HIV Testing Among Adults in Kenya. *AIDS Behav* 2020.

50. Menacho I, Sequeira E, Muns M, et al. Comparison of two HIV testing strategies in primary care centres: indicator-condition-guided testing vs. testing of those with non-indicator conditions. *HIV medicine* 2013; **14 Suppl 3**(100897392): 33-7.

51. Krakower DS, Gruber S, Hsu K, et al. Development and validation of an automated HIV prediction algorithm to identify candidates for pre-exposure prophylaxis: a modelling study. *Lancet HIV* 2019; **6**(10): e696-e704.

52. Joseph Davey DL, Wall KM, Kilembe W, et al. Difficult decisions: Evaluating individual and couple-level fertility intentions and HIV acquisition among HIV serodiscordant couples in Zambia. *PloS one* 2018; **13**(1): e0189869.

53. Damery S, Nichols L, Holder R, et al. Assessing the predictive value of HIV indicator conditions in general practice: a case-control study using the THIN database. *Br J Gen Pract* 2013; **63**(611): e370-7.

54. Manavi K, Damery S, Ryan R, et al. Targeted HIV screening in primary care; who should be tested? *HIV Medicine* 2012; **13**: 57.

55. Aim-Eusebi A, Prothon E, Majerholc C, Barger D, Yazdanpanah Y, Aubert J-P. The acceptability and effectiveness of a questionnaire for the identification of risk factors for HIV and hepatitis B and C: An observational study in general practice. *The European journal of general practice* 2018; **24**(1): 60-7.

56. Cayuelas Redondo L, Ruiz M, Kostov B, et al. Indicator condition-guided HIV testing with an electronic prompt in primary healthcare: a before and after evaluation of an intervention. *Sex Transm Infect* 2019; **95**(4): 238-43.

57. Feller D, Zucker J, Yin M, Gordon P, Elhadad N. Hiv risk assessment using longitudinal electronic health records. *Open Forum Infectious Diseases* 2017; **4**(Supplement 1): S419.

58. Leal L, Torres B, Leon A, et al. Predictive Factors for HIV Seroconversion Among Individuals Attending a Specialized Center After an HIV Risk Exposure: A Case-Control Study. *AIDS Res Hum Retroviruses* 2016; **32**(10-11): 1016-21.

59. Ayieko J, Kwariisima D, Koss CA, et al. Self-assessed HIV risk 3 years after HIV "test and treat" implemented in search. *Topics in Antiviral Medicine* 2017; **25**(1 Supplement 1): 365s.

60. Giovenco D, Pettifor A, MacPhail C, et al. Assessing risk for HIV infection among adolescent girls in South Africa: an evaluation of the VOICE risk score (HPTN 068). *J Int AIDS Soc* 2019; **22**(7): e25359.

61. Facente SN, Pilcher CD, Hartogensis WE, et al. Performance of risk-based criteria for targeting acute HIV screening in San Francisco. *PloS one* 2011; **6**(7): e21813.

62. Falasinnu T, Gustafson P, Gilbert M, Shoveller J. Validation of the denver HIV risk score for targeting HIV screening in Vancouver, British columbia. *Sexually Transmitted Infections* 2015; **91**(SUPPL. 1): A63.

63. Nelwan EJ, Isa A, Alisjahbana B, et al. Routine or targeted HIV screening of Indonesian prisoners. *International journal of prisoner health* 2016; **12**(1): 17-26.

64. Sampson LA, Miller WC, Leone PA. Evaluation of risk-score algorithms for the detection of HIV infection and syphilis in north carolina county jails. *Sexually Transmitted Infections* 2011; **87**: A210.

65. Kahle EM, Hughes JP, Lingappa JR, et al. An empiric risk scoring tool for identifying high-risk heterosexual HIV-1-serodiscordant couples for targeted HIV-1 prevention. *J Acquir Immune Defic Syndr* 2013; **62**(3): 339-47.

66. Okpokoro E, Osawe S, Daitiri R, et al. Validating HIV acquisition risk score using a cohort HIV exposed sero-negative persons in a discordant relationship in Jos, Nigeria, West Africa. *BMC Infectious Diseases* 2016; **16**(Supplement 2).

67. Smith DK, Pan Y, Rose CE, et al. A Brief Screening Tool to Assess the Risk of Contracting HIV Infection Among Active Injection Drug Users. *Journal of addiction medicine* 2015; **9**(3): 226-32.

68. Haukoos JS. The impact of nontargeted HIV screening in emergency departments and the ongoing need for targeted strategies. *Archives of internal medicine* 2012; **172**(1): 20-2.

69. Sibanda W, Pretorius P, Ozyer T, Carrington P. Development and validation of an HIV risk scorecard model. *2013 IEEE/ACM International Conference on Advances in Social Networks Analysis and Mining* 2013: 916-22.

70. Yumo HA, Kuaban C, Ajeh RA, et al. Active case finding: comparison of the acceptability, feasibility and effectiveness of targeted versus blanket provider-initiated-testing and counseling of HIV among children and adolescents in Cameroon. *BMC pediatrics* 2018; **18**(1): 309.

71. Caldwell DH, Jan G. Computerized assessment facilitates disclosure of sensitive HIV risk behaviors among African Americans entering substance abuse treatment. *Am J Drug Alcohol Abuse* 2012; **38**(4): 365-9.

72. Wahome E, Thiong'o AN, Mwashigadi G, et al. An Empiric Risk Score to Guide PrEP Targeting Among MSM in Coastal Kenya. *AIDS Behav* 2018; **22**(Suppl 1): 35-44.

73. Felsen UR, Cunningham CO, Heo M, Futterman DC, Weiss JM, Zingman BS. Expanded HIV Testing Strategy Leveraging the Electronic Medical Record Uncovers Undiagnosed Infection Among Hospitalized Patients. *Journal of acquired immune deficiency syndromes (1999)* 2017; **75**(1): 27-34.

74. Yumo HA, Ajeh RA, Beissner M, et al. Effectiveness of symptom-based diagnostic HIV testing versus targeted and blanket provider-initiated testing and counseling among children and adolescents in Cameroon. *PLoS One* 2019; **14**(5): e0214251.

75. Blumenthal J, Jain S, Mulvihill E, et al. Perceived Versus Calculated HIV Risk: Implications for Pre-exposure Prophylaxis Uptake in a Randomized Trial of Men Who Have Sex With Men. *J Acquir Immune Defic Syndr* 2019; **80**(2): e23-e9.
